# Compositional Changes of the High-Fat Diet-Induced Gut Microbiota upon Consumption of Common Pulses

**DOI:** 10.3390/nu13113992

**Published:** 2021-11-09

**Authors:** Tymofiy Lutsiv, Tiffany L. Weir, John N. McGinley, Elizabeth S. Neil, Yuren Wei, Henry J. Thompson

**Affiliations:** 1 Cancer Prevention Laboratory, Colorado State University, Fort Collins, CO 80523, USA; tymofiy.lutsiv@colostate.edu (T.L.); john.mcginley@colostate.edu (J.N.M.); elizabeth.neil@colostate.edu (E.S.N.); 2 Graduate Program in Cell and Molecular Biology, Colorado State University, Fort Collins, CO 80523, USA; tiffany.weir@colostate.edu; 3 Department of Food Science and Human Nutrition, Colorado State University, Fort Collins, CO 80523, USA; yuren.wei@colostate.edu

**Keywords:** lentil, chickpea, dry pea, common bean, pulses, high-fat diet, gut microbiome, cecal microbiota, mice

## Abstract

The gut microbiome is involved in the host’s metabolism, development, and immunity, which translates to measurable impacts on disease risk and overall health. Emerging evidence supports pulses, i.e., grain legumes, as underutilized nutrient-dense, culinarily versatile, and sustainable staple foods that promote health benefits through modulating the gut microbiota. Herein, the effects of pulse consumption on microbial composition in the cecal content of mice were assessed. Male mice were fed an obesogenic diet formulation with or without 35% of the protein component comprised by each of four commonly consumed pulses—lentil (*Lens culinaris* L.), chickpea (*Cicer arietinum* L.), common bean (*Phaseolus vulgaris* L.), or dry pea (*Pisum sativum* L.). Mice consuming pulses had distinct microbial communities from animals on the pulse-free diet, as evidenced by β-diversity ordinations. At the phylum level, animals consuming pulses showed an increase in Bacteroidetes and decreases in Proteobacteria and Firmicutes. Furthermore, α-diversity was significantly higher in pulse-fed animals. An ecosystem of the common bacteria that were enhanced, suppressed, or unaffected by most of the pulses was identified. These compositional changes are accompanied by shifts in predicted metagenome functions and are concurrent with previously reported anti-obesogenic physiologic outcomes, suggestive of microbiota-associated benefits of pulse consumption.

## 1. Introduction

The foods we eat directly contribute to our physical health and making better dietary choices can reduce disease risk [1,2]. However, current challenges in both nutrition and agriculture, including overpopulation that increases consumer demand and climate change that affects agricultural production, can reduce access to nutrient-dense whole foods. Concomitantly, reduced physical activity and a faster pace of life have created a worldwide milieu in which low-quality dietary patterns are considered “normal,” despite their contribution to an increasing burden of chronic diseases [3]. Improvement of dietary behaviors may benefit from the promotion of “novel superfoods” that provide ease of consumption, health benefits, and sustainability, and therefore fit well into the contemporary way of life [4]. Nevertheless, *everything new is a well-forgotten old*: pulses, a candidate category of superfoods, are nutritious, healthful, economical, ecologically sustainable, and gastronomically diverse foods that have been consumed by people across the globe for over ten millennia [5].

Pulses are leguminous dry grains, popular in culinary culture for their high protein and fiber content, making them a suitable alternative to foods of animal origin when compared to other plant foods that typically do not possess abundant protein [6]. With low amounts of fat and a high content of slowly digested carbohydrates, consumption of pulses lowers the glycemic index of a dietary pattern, creating a potential avenue to address chronic metabolic diseases, including diabetes and obesity [7]. Pulses also contain bioavailable micronutrients and bioactive compounds, such as potassium, iron, and vitamins as well as phenols, tannins, and flavonoids, which contribute to their health benefits [8]. The healthfulness of this staple food can manifest not only from consuming pulses as whole foods but also from the incorporation of pulses as a food ingredient [5]. Overall, regular pulse consumption has been associated with lower levels of inflammation and oxidative stress, improved weight management, lower risk of developing cardiovascular diseases and various types of cancer, and even increased longevity [9]. And yet, despite the extensive research on the benefits of pulses and the consequences of their consumption, an important question remains—*what are the mechanisms underlying pulse-induced health improvements?*

The gastrointestinal tract is a frontline mediator of effects that consumed foods exert on health and well-being, but there is more to this than just digestion and extraction of nutrients, absorption thereof, and removal of the wastes. Emerging research highlights the crucial role of the bidirectional effects between diet and gut microbiota on host health [10,11]. Intestinal bacteria contribute to the digestion of dietary components and synthesize vitamins, essential amino acids, short-chain fatty acids, and other bioactive compounds that affect not only local gut health but the overall health of the host [3,12]. A major part of the dietary impact on whole-body physiology may indeed be mediated by the gut microbiota, and such evidence is increasing rapidly [3,11,12,13,14]. Likewise, components of the diet stimulate or suppress specific groups of bacteria to modulate the composition of the gut microbiota and its downstream metabolite production. Identifying such eco-groups would allow the creation of a framework for describing the pattern of gut microbial changes in response to pulse consumption [15].

Despite many reports of the health benefits from the consumption of pulses, the data on their effects on gut microbiota composition and function are limited, and to our knowledge, a comparative analysis of effects on the gut microbiome among pulses has not been reported. Protein, polyphenols, and especially dietary fiber are major factors that drive changes in gut microbial communities and these components vary among pulses [6]. The work reported herein compares the impact of the four most consumed pulses—lentil (*Lens culinaris* L.), chickpea (*Cicer arietinum* L.), common bean (*Phaseolus vulgaris*, L.), or dry pea (*Pisum sativum* L.)—on microbiome composition and its predicted function in a murine model. The objective of this effort is to provide insights about pulse-associated microbial profiles and their potential role in maintaining health and well-being, as well as in disease pathogenesis, with the goal of identifying the gut microbial eco-groups affected by consumption of each pulse.

## 2. Materials and Methods

### 2.1. Experimental Design

This study was performed in accordance with the Colorado State University Institutional Animal Care and Use Committee (protocol 18-7746A). Anthropometric data from this study were initially reported in [16]. In the experiment presented here, cecal content from that study was subjected to a detailed analysis of the gut microbial ecosystem. Briefly, male 21–28-day-old NCI C57BL/6NCrl mice were purchased from Charles River Laboratories International, Inc., (Frederick, MD, USA). A total of 72 weanling mice were housed in solid bottomed polycarbonate rodent cages and maintained on a 12 h light/dark cycle at 27.5 ± 2 °C with 30% relative humidity with *ad libitum* access to the purified high-fat diet and distilled water. Mice were adapted to the purified diet formulation and animal husbandry routine during a 2-week timeframe (3 to 5 weeks of age). At 5 weeks of age, mice were randomized by body weight and assigned to their experimental diet groups (n = 6–8/group): The Control cohort was sustained on the high-fat diet (pulse-free); other diet groups consumed the high-fat formulation with lentil, chickpea, common bean, or dry pea powder replacing 35% of the protein content (Table 1). All the mice were fed their respective diets for 17 weeks. At 22 weeks of age, the experiment was terminated as animals were euthanized by cervical dislocation after isoflurane-induced anesthesia. Subsequently, the content of the ceca was harvested and snap-frozen in liquid nitrogen prior to DNA extraction. This study differs from other reports in that a single animal feeding experiment included the four pulses, while the vendor, shipment, and animal husbandry practices were the same, and thus their associated differences were minimized.

### 2.2. 16S rRNA Gene Library Preparation and Sequencing

DNA from cecal contents collected at necropsy was extracted using the QIAamp PowerFecal DNA kit (Qiagen, Germantown, MD, USA) following the manufacturer’s protocol, then checked for purity (260/280 and 260/230 ratios) and concentration via NanoDrop (Thermo Fisher Scientific, Waltham, MA, USA). Paired-end sequencing libraries of the V4 region of the 16S rRNA gene were constructed using the 515F-806R primer set according to the Earth Microbiome Project protocols [17], followed by sequencing using the MiSeq Reagent Kit v2 2 × 250 bp on an Illumina MiSeq instrument (Next-Generation Sequencing Facility at Colorado State University). 

### 2.3. Sequence Processing

The resulting forward and reverse paired-end sequence reads were processed with QIIME 2 platform, version 2021.2 [18]. Sequences were demultiplexed without Golay error correction and denoised by DADA2 pipeline [19]: each sequence pair was trimmed at 13 bp and truncated from 155 bp, checked for chimeras, and filtered for quality control. Taxonomy was assigned to amplicon sequence variants (ASVs) using Naive Bayes classifier [20,21] pre-trained on Greengenes (16S rRNA, version 13_8) marker gene reference database trimmed to the V4 domain (bound by the 515F/806R primer pair) with 99% sequence identity threshold [22]. The dataset was filtered to remove all features annotated as “mitochondria” and “chloroplast.” Based on the remaining features found in our data, a rooted phylogenetic tree was generated. The resulting dataset was used in two output forms—the raw abundance tables for ASVs and their respective taxonomic assignments.

### 2.4. Statistical and Bioinformatics Analyses

Data analysis was conducted in MicrobiomeAnalyst web-based platform [23,24] using the Marker-gene Data Profiling module therein. High-quality read counts ranged from 31,369 to 96,177 per sample. Features with less than two counts were automatically removed by pre-processing steps of MicrobiomeAnalyst’s integral Sanity Check. Data were further filtered for low abundance—a minimum of 10% samples contained at least four counts—as well as for the low variance—5% based on the inter-quantile range were removed. Total sum scaling (TSS) was performed to normalize the data. Consequently, the resulting datasets comprised an abundance table with 395 ASVs as well as an abundance table of their taxonomic composition mapped to 48 bacterial communities.

α-diversity was calculated from resulting ASV counts, analyzed via Chao1 and Shannon’s indices, and statistically compared using the Kruskal-Wallis non-parametric test on the feature level of taxonomy across the diet groups. Principal component analysis (PCoA) of β-diversity distances matrix based on Bray-Curtis index as well as Weighted and Unweighted UniFrac metrics were assessed on the feature level of ASV tables using permutational multivariate analysis of variance (PERMANOVA). 

Phyla level analyses were performed on abundance tables obtained from MicrobiomeAnalyst post filtering. Ratios were calculated by dividing raw Control counts from each phylum by their respective pulse-based groups. Pairwise comparison was performed in R studio (R version 4.1.1) using the Kruskal-Wallis and Dunn tests with the Benjamini-Hochberg method of *p*-values adjustment. 

The method of Random Forests Classification was used to determine a ranked list of the most important predictive bacterial taxa (biomarkers) able to discriminate among the diet groups [25]. The algorithm used 5000 trees and seven predictors with a randomness setting “on” to create a model trained on the feature level of the abundance data table of taxa. 

Bacterial biomarkers were also discovered using the linear discriminant analysis (LDA) effect size (LEfSe) method [26]. Briefly, this algorithm allows detection of differentially abundant taxa among the experimental groups using the Kruskal-Wallis test and then evaluates their relevance via LDA score. LEfSe was performed on the feature level of the taxa table using cutoffs at less than 0.05 for the FDR-adjusted *p*-value and beyond the absolute value of 2.0 for the logarithmic LDA score. 

Variation of taxonomic abundance related to the diet group was visualized on a heatmap after Ward’s hierarchical clustering algorithm based on Minkowski distances. Feature level was used for the analysis of the taxa-assigned abundance table. 

Correlation analysis was performed to build a correlation network between bacterial pairs using Sparse Correlations for Compositional data (SparCC) algorithm [27] with 100 permutations in MicrobiomeAnalyst. Only bacteria that passed the correlation threshold 0.4 and 0.7, as well as *p*-value threshold 0.05, were included in the results. 

Functional attributes of the identified microbial communities were predicted using Phylogenetic Investigation of Communities by Reconstruction of Unobserved States 2 (PICRUSt2) pipeline, version 2.4.1 [28]. With the previously obtained ASVs dataset as an input, PICRUSt2 performs phylogenetic placement by aligning ASVs to the reference 16S sequences (HMMER, www.hmmer.org) and incorporating them into the reference tree (evolutionary placement algorithm (EPA)-NG and genesis applications for phylogenetic placement analysis (GAPPA) [29,30], followed by the hidden-state prediction of gene families (castor R package [31]) and, finally, generation of metagenomic predictions and tabulation of pathways’ inferences and abundances (Minimal set of Pathways (MinPath) [32] and MetaCyc [33]. Statistical analysis of taxonomic and functional profiles (STAMP) software, version 2.1.3 (Robert Beiko, Halifax, NS, Canada), was used to analyze and visualize PICRUSt2 output data [34]. In brief, the pulse-free Control group and samples from pulse-based diet groups were compared using Welch’s two-sided *t*-test with 0.95 Welch’s inverted CI method and Benjamini-Hochberg FDR multiple test corrections method. Changes in pathways with effect size below 20% for difference between proportions and above 1.5-fold ratio of proportions were considered relevant.

## 3. Results

### 3.1. Overall Response to Pulse Consumption

**Figure 1:**
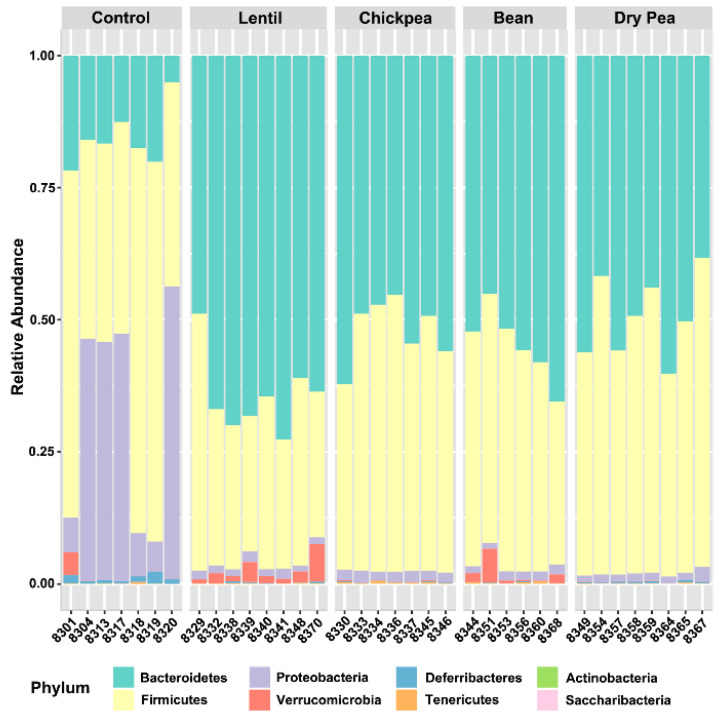
Relative abundance of identified Phyla across individual samples per each diet formulation.

After filtering and normalization steps (described in Section 2.1), the final dataset contained 395 ASVs that were mapped to 48 bacterial groups at different taxonomic levels. The bacteria were distributed among eight phyla (Figure 1). 

Bacteroidetes and Firmicutes were the most abundant across all diet groups, followed by Proteobacteria. The pulse-based diet groups had a 2.5–4.9-fold increase in the Bacteroidetes abundance in comparison to the pulse-free Control, with the Bean diet having the lowest and the Lentil having the highest fold change. The latter two pulse groups also had a 1.7- and 1.4-fold decrease in Firmicutes levels, respectively, compared with the pulse-free Control diet, whereas Chickpea and Dry Pea diet groups had comparable amounts thereof. The Firmicutes to Bacteroidetes ratio was 3.3 for the pulse-free Control and ranged from 0.48 to 0.97 among the pulses, with the Lentil group exhibiting the lowest value. Proteobacteria was the third most abundant phylum, especially in the pulse-free Control diet (32% of the total, represented predominantly by bacteria from the Deltaproteobacteria class). The abundance of Proteobacteria decreased 16–29.2-fold in the pulse-based diets. Both Bean and Lentil groups were also distinguished by a higher abundance of Verrucomicrobia, represented by *A. muciniphila* (2.1- and 5.2-fold increase, respectively, versus the pulse-free Control). Deferribacteres were 2.9–19.5-times more abundant, whereas Tenericutes were less present in the control diet (Table 2). Interestingly, unlike the pulse diet groups, the pulse-free Control group did not have any representatives of the Saccharibacteria phylum.

**Figure 2:**
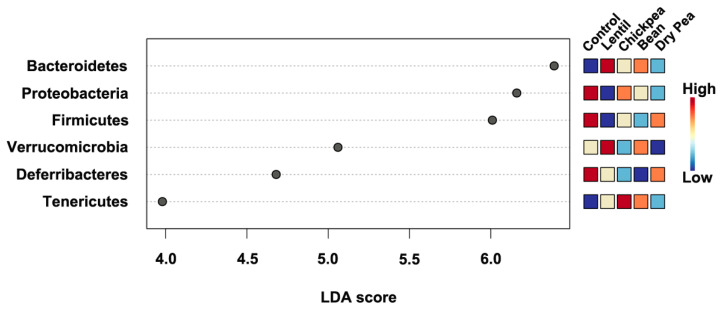
The linear discriminant analysis (LDA) of effect size (LEfSe) between the diet groups at the phylum level. All represented phyla were statistically significant (LDA score > |2.0|). Relative abundance per diet group is represented on the heatmap to the right.

LEfSe indicated that all identified phyla, except for Saccharibacteria and Actinobacteria, were statistically significantly different between the diet groups with Bacteroidetes, Proteobacteria, and Firmicutes scoring above 6.0 (Figure 2). However, according to the Kruskal-Wallis and Dunn tests pairwise comparison results, Saccharibacteria were differential across several diets (Table 2). 

### 3.2. Effects on α-Diversity

**Figure 3:**
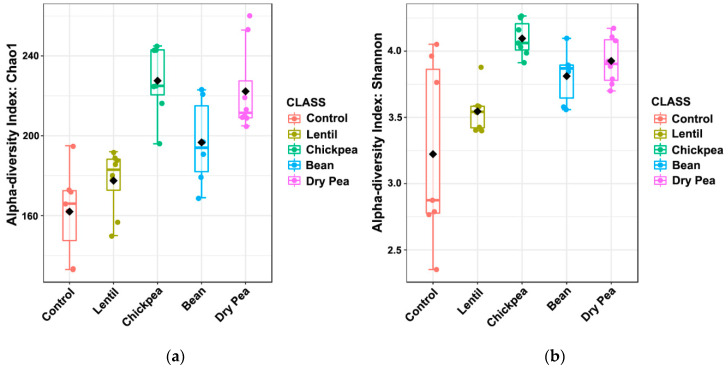
The boxplots of α-diversity metrics of the cecal microbiota across diet groups at the ASVs level: (**a**) α-diversity measured with the Chao1 index indicating differences in richness; Kruskal-Wallis test statistic = 24.501, *p*-value = 6.338 × 10^−5^; (**b**) α-diversity measured with the Shannon index accounting for differences in richness and evenness; Kruskal-Wallis test statistic = 18.41, *p*-value = 1.026 × 10^−3^.

The microbial communities maintained by each of the diets were analyzed for their intragroup dissimilarity. The diet groups were statistically different in terms of their α-diversity distributions (Figure 3). Estimated species richness (Chao1) and both community richness and evenness (Shannon’s index) had *p*-values < 0.001 and <0.01, respectively, using Kruskal-Wallis testing. The pulse-free Control group showed a tendency to be the least intra-individually diverse. The most diverse microbial communities were found in the Chickpea and the Dry Pea groups.

### 3.3. Effect on β-Diversity

**Figure 4:**
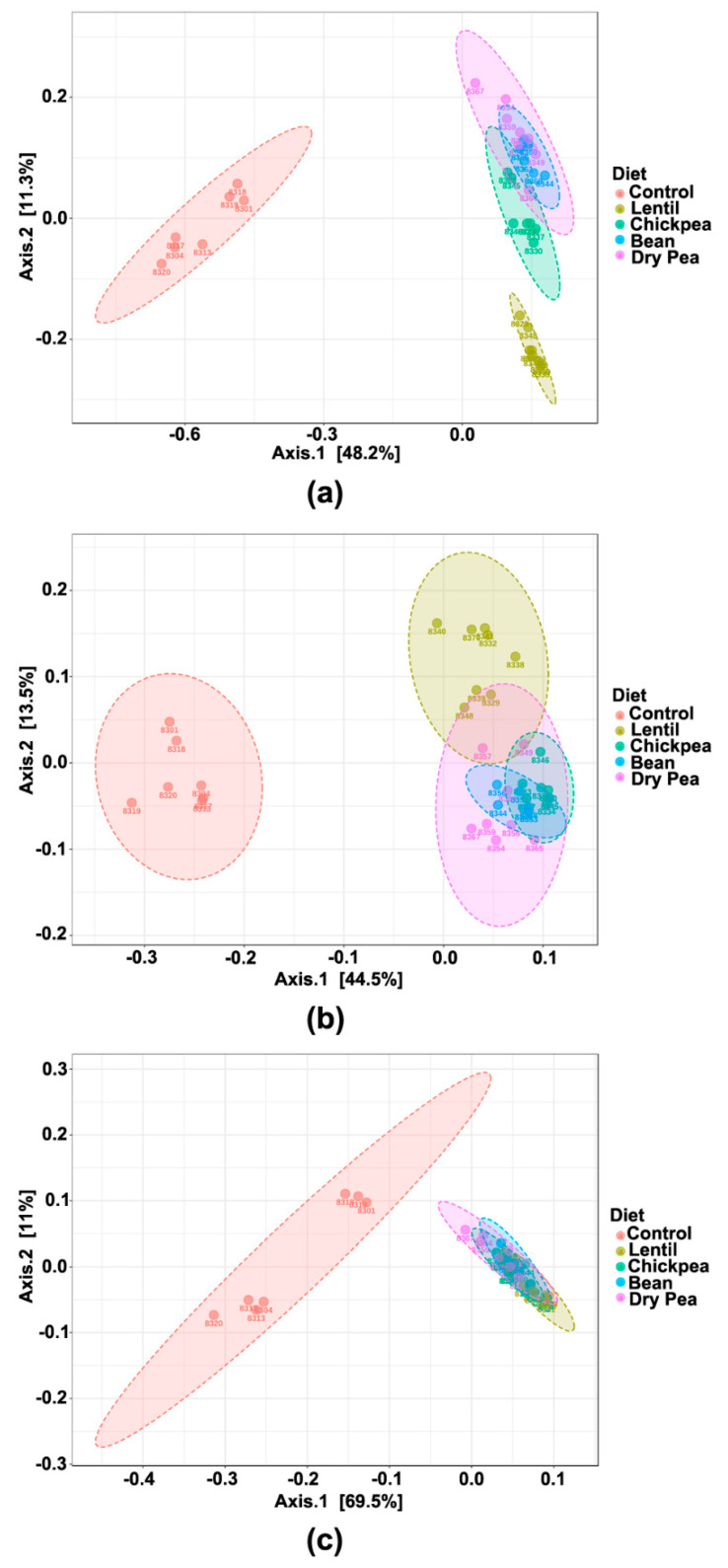
Measures of β-diversity across the diet groups at the ASVs level plotted in the Principal component analysis (PCoA) in 2D. Axes 1 and 2 represented principal components 1 (PC1) and 2 (PC2), respectively. Samples are colored and grouped into ellipses by their corresponding diet group. Differences in the β-diversity were tested by the permutational multivariate analysis of variance using distance matrices (PERMANOVA): (**a**) Bray-Curtis distances were used to explain β-diversity across diet groups. PERMANOVA *F*-value = 16.312; *R*^2^ = 0.67792; *p*-value < 0.001; (**b**) Unweighted UniFrac distances were used to explain β-diversity across diet groups. PERMANOVA *F*-value = 13.642; *R*^2^ = 0.63772; *p*-value < 0.001; (**c**) Weighted UniFrac distances were used to explain β-diversity across diet groups. PERMANOVA *F*-value = 20.808; *R*^2^ = 0.72862; *p*-value < 0.001.

The diet-induced bacteria were also analyzed for their intergroup dissimilarity based on their ASV values as reflected in β-diversity with statistical testing using PERMANOVA. PCoA based on Bray-Curtis indices shows that the pulse-free Control group separates completely from the pulse groups along the PC1 (Figure 4a). The latter tend to cluster together, with the Lentil group showing separation along PC2. Plotting unweighted UniFrac distances confirmed this separation (Figure 4b, *p*-value < 0.001). This metric incorporates phylogenetic ties and focuses on the absence and presence of the taxa, making it more sensitive towards rare and low-abundant organisms. The pulse-free Control separates from the pulse groups along the PC1 axis, and the Lentil group, to a much smaller extent, differs the most from the rest of the pulses along PC2. Such differences between Lentil and the rest of the pulse-based diet groups correspond to the Kruskal-Wallis results of the phyla abundances differences (Table 2). However, when β-diversity was assessed using Weighted UniFrac distances (Figure 4c), which emphasize the impact of the most abundant bacteria in the community on the qualitative differences among the diet groups, the pulse-free Control separates from the pulse groups, but without distinction among pulse groups (*p*-value < 0.001). These findings point to the prominent similarity of the microbial communities among the pulse-based diet groups in terms of the most abundant and dominant bacteria in the gut.

### 3.4. Which Bacteria Are the Major Players in Accounting for Differences Due to Pulse Consumption?

**Figure 5:**
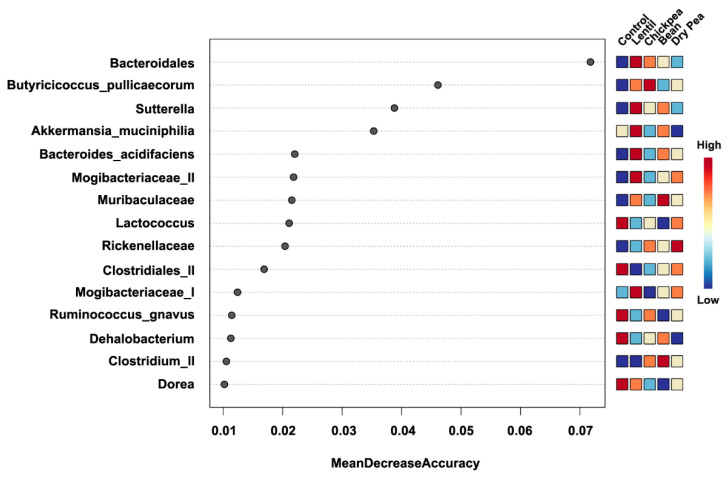
Top 15 bacteria to discriminate between the diet groups predicted by the Random Forest algorithm. Features are ranked by their contributions to classification accuracy (Mean Decrease Accuracy). Relative abundance per diet group is represented on the heatmap to the right.

The Random Forest supervised learning algorithm was performed to determine the most important predictive microbial communities (represented by their lowest taxonomic rank that was assigned) to classify the diet groups (Figure 5). The top 10 most influential biomarkers that drive the differences between the diets mapped to unclassified Bacteroidales, *B. pullicaecorum*, *Sutterrella*, *A. muciniphila*, *B. acidifaciens*, Mogibacteriaceae (II), Muribaculaceae, *Lactococcus*, Rikenellaceae, and Clostridiales (II).

### 3.5. Diet-Specific Microbial Ecosystems

**Figure 6:**
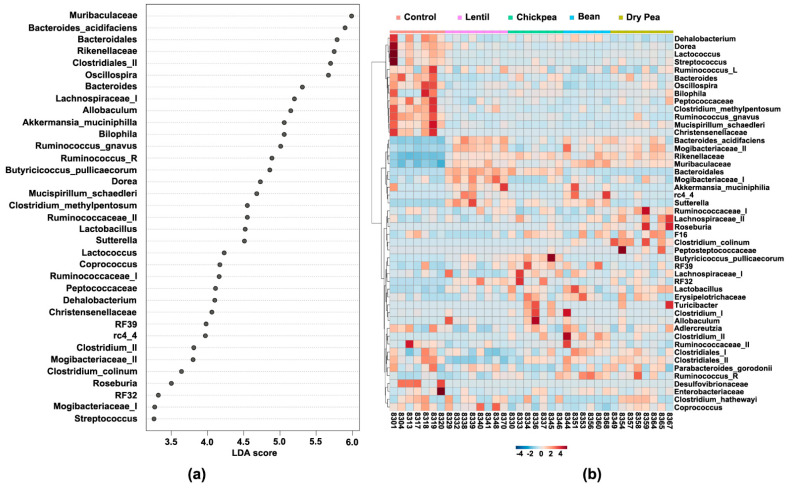
Differential abundance analysis of cecal microbiota across the diet groups: (**a**) The linear discriminant analysis (LDA) of effect size (LEfSe) between the diet groups at the feature level. All represented bacteria were statistically significant (LDA score > |2.0|); (**b**) Heatmap representing hierarchical clustering analysis results at the feature level. Clusters were organized by the diet group factor using Ward’s algorithm and Minkowski’s distance measure.

In order to ascertain which bacteria are statistically different across the diet groups, LEfSe was performed at the feature level on the compositional abundance dataset. Overall, there were 35 microbial groups that were statistically different among all the diets (Figure 6a). LEfSe was supplemented with the hierarchical clustering analysis plotted on a heatmap so as to elucidate how the cecal bacteria were distributed across the samples (Figure 6b). These tests allow the determination of the most differential bacteria in the gut microbiome as well as distribution thereof across the diet groups. Next, pairwise LEfSe contrasting Control versus each pulse separately was performed to identify pulse-specific microbial changes. Finally, LEfSe was also performed on the dataset containing pulse-based diet groups only to complete the overall comparative picture of differential microbial communities—18 bacteria were significantly distinct in their abundance amongst the pulse-based diets. Results derived from such LEfSe-based analyses are summarized in Table 3. 

As one can see, within each of the groups, a number of selected bacteria were the same for every pulse type (Table 3). Abundances of Muribaculaceae, *B. acidifaciens*, Rikenellaceae, *Allobaculum*, *B. pullicaecorum*, *Sutterella*, Mogibacteriaceae (II), *rc4 4* (of Peptococcaceae), and RF32 (of Alphaproteobacteria) were commonly enhanced in pulse-based diets compared with the Control. The dietary effect of pulses can also be evaluated from the perspective of the microbiota that were decreased, i.e., taxa with significantly lower abundance in the pulse-containing versus the Control diet: *Oscillospira*, *R. gnavus*, *M. schaedleri*, *Dorea*, *C. methylpentosum*, *Lactococcus*, Peptococcaceae, Christensenellaceae, and *Streptococcus*. Finally, no statistically significant differences were detected in the abundances of *Adlercreutzia, Bilophila*, Clostridiales (I), *C. hathewayi*, *Coprococcus*, Desulfovibrionaceae, Enterobacteriaceae, Erysipelotrichaceae, F16, *P. gordonii*, Ruminococcaceae (I), and *Ruminococcus* (of Lachnospiraceae) between each pulse-based diet and Control group. Interestingly, unclassified species of *Coprococcus* and Ruminococcaceae (I) appeared significantly differential in the LEfSe results across all tested diet groups (Figure 6a) but were assigned as unaffected by the pulse consumption due to their lack of statistical significance in the pulse-specific and pairwise comparison against the Control group analyses (Table 3). Such discrepancy may be due to their uneven distribution across all the tested samples (Figure 6b). 

**Figure 7:**
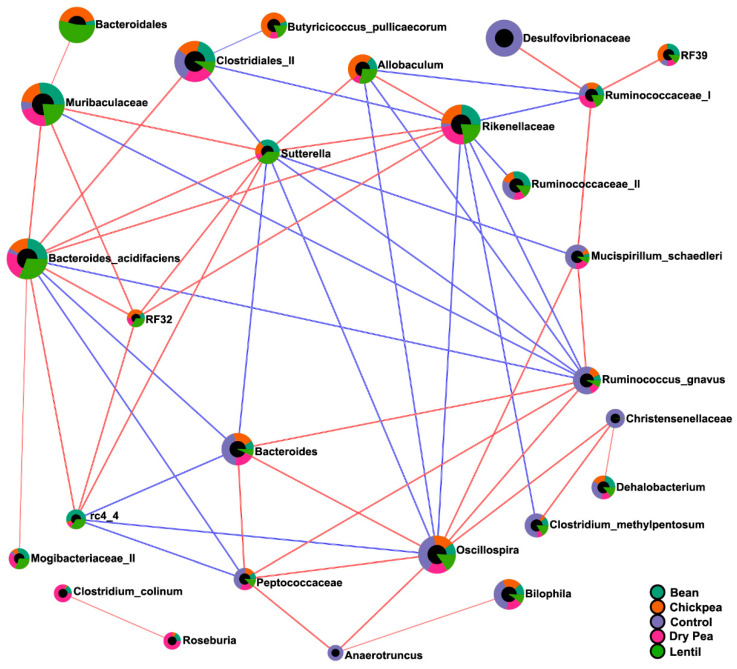
Correlation network of cecal microbiota across the diet groups. Each node represents a taxon, colored by the mean abundance in a respective diet group. Nodes are connected by the edges representing correlations and their value between taxa pairs: blue indicates negative, while red indicated positive correlation. A correlation network was generated using the SparCC algorithm with 100 permutations, depicting only taxa that met the *p*-value threshold 0.05 and the correlation threshold 0.7.

Finally, correlation analysis was performed on the compositional abundance dataset to uncover potential co-occurrence patterns amongst bacteria across all the diet groups, and the results thereof were presented in the form of a correlation network (Figure 7). 267 bacterial pairs had a correlation coefficient above 0.4 (Supplementary Table S1), out of which 57 pairs strongly correlated with a SparCC coefficient above 0.7. Previously identified eco-groups tend to significantly correlate with each other—the pulse-enhanced bacteria correlated positively with each other and negatively with those pulse-suppressed, and vice versa. 

The pulse-based diets exhibited overall similarity in their gut microbial composition, especially in the identified common eco-groups; however, some differences were still present as determined by excluding the Control group from the dataset and subjecting the remaining data to LEfSe analysis (Table 3, marked *). *The Lentil cohort*, which differed the most from the rest of the pulse-based diets, was characterized by a higher abundance of unclassified Bacteroidales and *B. acidifaciens*, *A. muciniphila*, *Sutterella*, both unclassified Mogibacteriaceae, and even suppressed *Dorea.* Compared with the Control, this diet group significantly decreased the presence of *Anaerotruncus*, *Dehalobacterium*, *Bacteroides*, and unassigned Clostridiales (II), exhibiting the lowest abundance of the latter two, unlike the other pulses. *The Chickpea group* was typified by an enhanced abundance of unclassified Lachnospiraceae (I), *Lactobacillus*, RF39 of Mollicutes, and unassigned *Clostridium (II)* of Clostridiaceae family compared with the Control, whereas from the rest of the pulses, Chickpea differed by the higher counts of unclassified *Bacteroides*, *Bilophila*, and especially *B. pullicaecorum*. *The Bean-based diet* group significantly enhanced *A. muciniphila*, unassigned Bacteroidales, *Ruminococcus* of Ruminococcaceae, and exceptionally unclassified *Clostridium* sp. *(II)* of Clostridiaceae compared to the pulse-free Control. The latter, together with the *rc4 4* of the family Peptococcaceae, as well as unaffected *Dehalobacterium* and Ruminoccoccaceae (II), had the highest relative abundance even next to the pulse-based cohorts. Finally, *the Dry Pea*-induced gut microbiome was distinctly represented by *Lactobacillus*, Lachnospiraceae (II), and especially by highly correlated *C. colinum* and *Roseburia*, among the enhanced from the Control group bacteria, as well as Ruminococcaceae (II), *Dehalobacterium*, and *Anaerotruncus* on top of pulse-suppressed eco-group. Furthermore, from the rest of the pulses, the Dry Pea cohort has significantly the highest abundance of *M. Schaedleri* and unclassified Clostridiales (II). Additional molecular studies are needed to determine whether these differences translate into the altered cell and metabolic signaling within the host.

### 3.6. Pulse-Predicted Microbial Function

**Figure 8:**
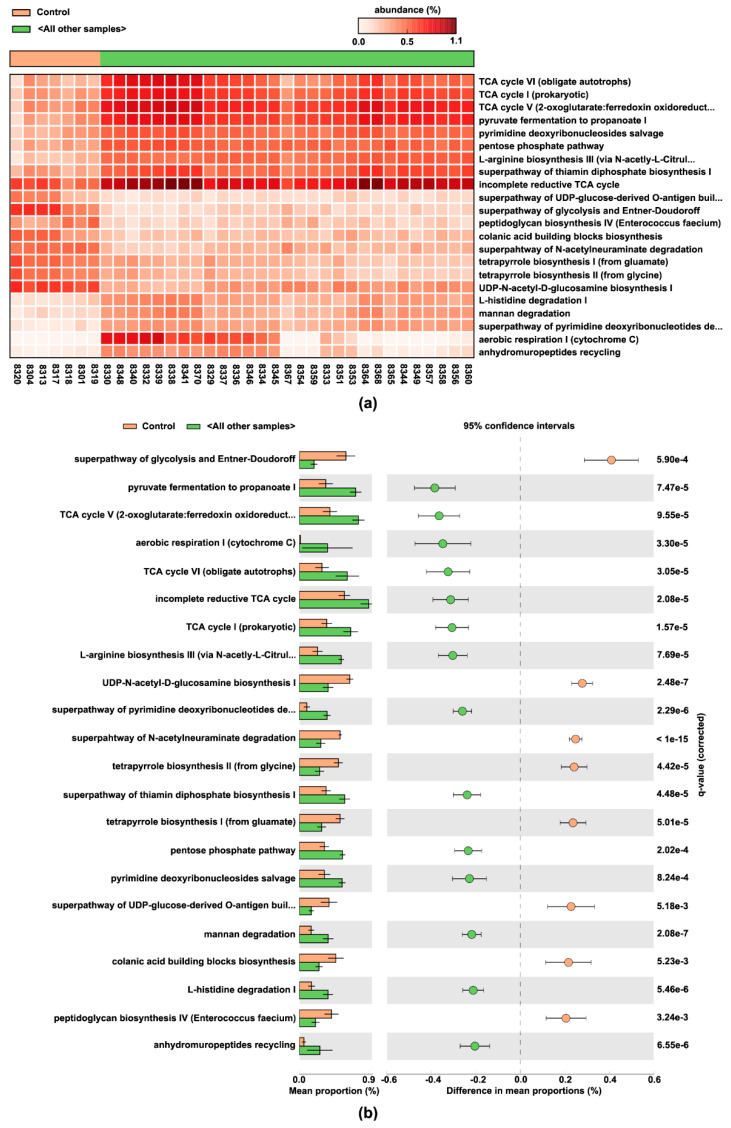
PICRUSt2 results indicating differential pathways between the pulse-free and pulse-based diet groups. Twenty-two out of 179 significant pathways with effect size over 20% for the difference between proportions and over 1.5-fold for the ratio of proportions are presented here. Pulse-free diet (“Control”) is indicated in beige, while pulse-based diet samples (“All other samples”)—in green: (**a**) Heatmap plot indicating the abundance of pathways assigned to each sample. (**b**) Extended error bar plot indicating the mean proportion of pathways assigned to each group, difference between them, and corrected *p*-value (*q*-value) of each.

Lastly, the metagenomic changes of microbial functions induced by each diet formulation were predicted and functionally annotated by the PICRUSt2 pipeline. Using Welch’s two-sided *t*-test with Benjamini-Hochberg FDR corrections, 179 statistically significant differential metabolic pathways were identified (Supplementary Figure S1), out of which 22 had effect size over 20% for the difference between proportions, and over 1.5-fold for the ratio of proportions (Figure 8).

As a result, pulses most effectively upregulated pyruvate fermentation to propanoate I, the tricarboxylic acid (TCA) cycle V (2-oxoglutarate: ferredoxin oxidoreductase), aerobic respiration I (cytochrome c), TCA cycle VI (obligate autotrophs), incomplete reductive TCA cycle, TCA cycle I (prokaryotic), L-arginine biosynthesis III (via N-acetyl-L-citrulline), super-pathway of pyrimidine deoxyribonucleotides de novo biosynthesis, super-pathway of thiamin diphosphate biosynthesis I, pentose phosphate pathway, pyrimidine deoxyribonucleosides salvage, mannan degradation, L-histidine degradation I, and anhydromuropeptides recycling among others. In contrast, the pulse-free Control group exhibited upregulation of super-pathway of glycolysis and Entner-Doudoroff, UDP-N-acetyl-D-glucosamine biosynthesis I, super-pathway of N-acetylneuraminate degradation, tetrapyrrole biosynthesis I (from glutamate) and II (from glycine), super-pathway of UDP-glucose-derived O-antigen building blocks biosynthesis, colonic acid building blocks biosynthesis, and the peptidoglycan biosynthesis IV (*Enterococcus faecium*).

## 4. Discussion

Considerable effort has been expended to identify dietary habits associated with human health benefits and, most recently, on the determination of the microbial ecosystems maintained in the gut by disease-associated versus health-promoting dietary patterns [15,35]. Pulses are unique among other widely consumed categories of food in two important aspects relative to human health: (1) the one-to-one-ratio of fiber and protein per unit weight in the absence of a significant amount of lipids, and (2) the fact that pulses are generally consumed as a whole food, but when they are consumed as an ingredient the whole cooked seed can be milled and freeze-dried such that the ingredient powder (flour) is equivalent to the whole food [5,6]. Therefore, we focused our data-driven approach on elucidating the microbial ecosystem characteristic of a level pulse consumption that exerts anti-obesogenic activity [16]. The null hypothesis that no differences would exist in microbiota composition across the five diet groups was rejected—perhaps best evidenced by highly significant differences in α- and β-diversity (Figure 3 and Figure 4). Those differences are easily visualized at the phylum level (Figure 1 and Figure 2, Table 2). The results of the unweighted and weighted UniFrac analyses (Figure 4) led us to conceptualize the identification of a pulse-induced ecosystem consisting of three eco-groups: one enhanced by all pulses, irrespective of pulse-type, an eco-group that was suppressed by pulse consumption, i.e., the taxa that were predominant in the pulse-free control diet, and an eco-group of microbiota unaffected by the pulse consumption, i.e., abundance was not statistically different between pulse-free and pulse-containing diets. It was achieved by performing differential analyses using the LEfSe method on the same dataset but with a different focus (Table 3 and Table 4) and confirmed by the cluster and correlation analyses (Figure 6b and Figure 7). 

We discovered nine microbial communities in a high-fat diet, the abundance of which was increased by pulse consumption, irrespective of type (Table 4). Among these, Bacteroidales, which include Muribaculaceae, *B. acidifaciens*, and Rikenellaceae scored the highest LDA and were the most representative of the pulse-based diets (Figure 6). They were also the main representatives of Bacteroidetes that accounted for the decrease in the Firmicutes/Bacteroidetes ratio in the pulse groups. Mostly known as *S24-7* [36], the Muribaculaceae family is one of the most dominant murine gut bacteria, known for its ability to degrade complex dietary carbohydrates [37]. Their abundance was shown to be decreased in obese mice [38] and significantly increased under high fiber [39]. In this experimental setting, Muribaculaceae were the most abundant in the Bean-based diet compared to the other groups, whereas in the pulse-free Control they were the least abundant. 

The other nine bacterial groups had significantly lower abundance in the pulse-containing versus the Control diets (Table 3). Most of these bacteria have been connected to obesity development and a higher risk of metabolic diseases [40,41,42,43]. Reduction in their abundance herein is a promising discovery in the context of anti-obesogenic and other reported health benefits of pulses. *Mucispirillum schaedleri* has been reported to be a marker of a high-fat diet [44]; it also positively correlates with serum levels of leptin and body fat [45] and decreases upon dietary treatment of non-alcoholic steatohepatitis [46,47]. *Ruminococcus gnavus* has been associated with gut dysbiosis and inflammatory diseases, such as inflammatory bowel disease, spondylo-arthritis, eczema, and pouchitis, but also allergic, coronary artery, and obesity-related diseases [48,49,50,51,52,53,54]. Interestingly, *R. gnavus* showed inverse relationships with *A. muciniphila* in the intestinal epithelium during the progress of the inflammatory bowel disease, despite both of them being the mucolytic bacteria [55]. Other bacteria that decreased upon pulse consumption, possibly owing to casein reduction, are *Lactococcus* sp. [56,57,58].

The abundance of eleven bacteria was statistically unchanged across diet groups based on differential abundance analyses and thus were deemed indifferent to the pulse consumption. Amongst these are bacteria that belong to the phyla that were also not significantly differential across the diets according to LEfSe (Figure 2). Interestingly, Desulfovibrionaceae and their genus *Bilophila* were visually representative bacteria of the pulse-free Control but did not reach significance in the Control vs. pulses LEfSe results possibly due to the high variation in abundance across the Control samples. However, while Desulfovibrionaceae correlated positively with members of pulse-suppressed eco-group members, such as *C. methylpentosum*, *R. gnavus*, *M. schaedleri*, and *Oscillospira*, pulse-enhanced *Allobaculum*, *B. acidifaciens*, and Rikenellaceae exhibited moderately negative relationships with this family (Table S1). Similarly, correlation analysis allows inference of associations of other pulse-indifferent bacteria, such as *Adlercreutzia*, *Bilophila*, *C. hathewayi*, *Coprococcus*, F16, *P. gordonii*, Ruminococcaceae (I), and *Ruminococcus* (of Lachnospiraceae family) with microbiota that were suppressed by pulses (Table S1). 

Metagenomic functional predictions demonstrated that pulse-based diets differ from the control diet by 82 upregulated pathways and 97 downregulated bacterial pathways (Figure S1). Propanoate production, which scored the highest, is associated with lowering energy intake and protecting from obesity and cancer development via reduced lipogenesis, circulating cholesterol, and inflammatory response [59,60,61]. Pulse consumption also predicted synthesis of vitamins and organic cofactors, such as thiamin, heme *b*, pyridoxal 5′-phosphate, flavin, folic acid (via 6-hydroxymethyl-dihydropterin diphosphate), NAD, phospho-pantothenate, coenzyme A. These data imply additional health benefit of pulse consumption associated with vitamin production [62]. Biosynthesis of a number of amino acids, e.g., L-methionine, L-phenylalanine, L-tyrosine, is predicted to be enhanced, whereas L-ornithine, L-lysine, L-threonine, L-tryptophan, L-isoleucine, L-valine biosynthesis was predicted to be reduced. Moreover, L-histidine, L-leucine (with additionally reduced biosynthesis), L-glutamate degradation pathways were also upregulated in the pulse-associated bacteria. L-arginine biosynthesis showed mixed results: citrulline-driven pathway was enhanced, whereas its other anabolic pathways were inhibited. This complex array of predictions requires further investigation to measure pulse-specific effects via metabolomics. Other predictions about the impact of pulsed consumption included: (1) reduced biosynthesis of colonic acid building blocks, and phosphatidylglycerols, and (2) stimulated degradation of IMP, glycerol, and several carbohydrates, such as mannan, xylose, rhamnose, lactose, galactose. In contrast, pathways associated with carbohydrate synthesis, especially those associated with building blocks for bacterial membrane components, were predicted to be enriched in the pulse-free Control microbiome. Additionally, pulse consumption was predicted to enhance the degradation of glucose, whereas the Control diet was predicted to upregulate glucose-producing pathways in the gut microbiota. These microbiota-driven effects could possibly contribute to the reduced glycemic response associated with pulse consumption [63]. 

As noted above, we have previously reported that each type of pulse that was investigated induced a significant reduction in adiposity in the absence of an effect on growth rate [16]. To our knowledge, this is the first in-depth comparative analysis of the nature of such differential effects of these pulses on the microbial ecosystem. In addition, the work focuses on the microbiome in cecal content rather than excreted fecal pellets. In so doing the ability to detect differences in populations of obligate anaerobes, a type of commensal microorganism thought to have a considerable impact on the health of the host, was maximized, while the variation that may occur, when excreted fecal pellets are evaluated, was reduced [64]. Finally, a high-fat diet was used to mimic an obesogenic dietary pattern, whereas its pulse-based modifications matched macronutrient content with the former but varied in their source of dietary protein and carbohydrate: casein-derived protein and carbohydrate from refined sources, i.e., mono- and disaccharides, as well as corn starch and cellulose, in the Control were replaced by 35% with the respective pulse protein and its associated carbohydrate. Thus, this approach modeled four different dietary patterns from populations around the world that preferentially consume one type of pulse. Despite these strengths, mice are not people, and this will remain a limitation in any study of the effects of diet on gut microbial composition and function conducted in preclinical models [65,66]. Nonetheless, the control afforded by such preclinical models allows for the deconstruction of complex observations made in human populations which is the counterbalancing strength of the work reported herein. Furthermore, efforts were made to minimize the impact of recognized sources of variation in preclinical studies of diet and the gut microbiome. 

The work presented here has empirical value in clarifying that macronutrient-matched pulse-free versus pulse-containing diets can be expected to differentially impact the gut microbiome and that pulse-type is an important variable that needs to be considered in both the design and interpretation of human studies. However, while advancing the concept of a pulse-induced ecosystem, it should be anticipated that the three components of the ecosystem will be populated by different microbial taxa in results emerging from different studies, and particularly those reported by different laboratories, in part due to factors such as a source of animals and differences among studies in animal husbandry practices [67]. Our data imply that the positive effects of pulse consumption on health may, in part, be mediated by the gut microbiota based on the magnitude of their pulse-driven community differences despite the obesogenic environment. Future work needs to address the dearth of knowledge about the dietary components that drive the differences reported herein and about how the pulse-induced microbial ecosystem exerts its effects on the host. It is likely that multi-omics approaches will provide the greatest insights as preclinical models are used to deconstruct the mechanisms underlying pulse-induced health benefits, and in so doing, facilitate the design of precision nutrition approaches to health promotion and disease prevention in human populations.

## Figures and Tables

**Table 1:** Formulations of experimental diets.

Ingredient	*High-Fat Pulse-Free Control ^1^*	*High-Fat Pulse-Based Diet ^1,5^*
	g/100 g	g/100 g
*Solka-Floc*	6.46	0
*Pulse Crop ^5^*	0	40
*Casein*	25.85	17.05
*Cerelose*	16.15	0
*Sucrose*	8.89	0.3
*Vitamin mix ^2^*	1.29	1.29
*DL-Methionine*	0.39	0.39
*L-Tryptophan ^3^*	0	0.01
*Choline bitartrate (41% choline)*	0.26	0.26
*Mineral mix ^4^*	5.82	5.82
*Soybean oil*	3.23	3.23
*Lard*	31.66	31.66

^1^ Experimental diets modified from the original diet formulations; ^2^ Dyets #310025 AIN-93G vitamin mix; ^3^ Sigma T0254-25G L-Tryptophan; ^4^ Dyets #210025 AIN-93G mineral mix; ^5^ Dumas nitrogen(%) of complete diet mixture before oil was added: high fat, 6.3; chickpea, 5.8; dry pea, 6.3; lentil, 6.5; kidney bean, 7.0.; For each pulse treatment group, the whole pulse was cooked and processed with the leachate, freeze-dried, and homogenized into a fine powder.

**Table 2:** Relative abundances of phyla per each diet group.

Phyla	*Control, %*	*Lentil, %*	*Chickpea, %*	*Bean, %*	*Dry Pea, %*
Actinobacteria	0.020	0.008	0.016	0.023	0.017
Bacteroidetes	15.533	64.941 ^1,^***	52.212 ^1,^*	54.500	49.689 ^1,^*
Deferribacteres	0.966	0.166 ^1,^**	0.109 ^1,^**	0.071 ^1,^***	0.294
Firmicutes	51.189	30.838 ^1,^*	45.475	41.992	48.367 ^2,^*
Proteobacteria	31.654	1.496 ^1,^**	1.898	1.545 ^1,^***	1.484 ^1,^**
Saccharibacteria	0.000	0.001	0.013 ^1,^*^; 2,^*	0.012	0.015 ^1,^*^; 2,^*
Tenericutes	0.089	0.100	0.213 ^1,^*	0.239	0.106
Verrucomicrobia	0.549	2.450	0.064 ^1,^*^; 2,^*	1.619	0.026 ^1,^*^; 2,^*

^1^ Significantly different from the Control group; ^2^ Significantly different from the Lentil group; Statistically significant phyla were determined by the Kruskal-Wallis test with * *p*-value < 0.05; ** p-value < 0.01; *** *p*-value < 0.001. *p*-values adjusted with the Benjamini-Hochberg method.

**Table 3:** Bacterial abundance changes across the diet groups compared with Control.

Cecal Bacteria	*Lentil*	*Chickpea*	*Bean*	*Dry Pea*
*Adlercreutzia*	≈	≈	≈	≈
Akkermansia muciniphila *	↑	≈	↑	≈
*Allobaculum*	↑	↑	↑	↑
*Anaerotruncus*	↓	↓	≈	↓
Bacteroidales *	↑	↑	↑	—
Bacteroides acidifaciens *	↑	↑	↑	↑
Bacteroides *	↓	≈	↓	≈
Bilophila *	≈	≈	≈	≈
Butyricicoccus pullicaecorum *	↑	↑	↑	↑
*Christensenellaceae*	↓	↓	↓	↓
*Clostridiales (I)*	≈	≈	≈	≈
Clostridiales (II) *	↓	≈	≈	≈
Clostridium colinum *	—	≈	—	↑
*Clostridium hathewayi*	≈	≈	≈	≈
*Clostridium (I)*	≈	≈	—	≈
Clostridium (II) *	—	↑	↑	≈
*Clostridium methylpentosum*	↓	↓	↓	↓
*Coprococcus*	≈	≈	≈	≈
Dehalobacterium *	↓	≈	≈	↓
*Desulfovibrionaceae*	≈	≈	≈	≈
Dorea *	↓	↓	↓	↓
*Enterobacteriaceae*	≈	≈	≈	≈
*Erysipelotrichaceae*	≈	≈	≈	≈
*F16*	≈	≈	≈	≈
*Lachnospiraceae (I)*	≈	↑	≈	≈
*Lachnospiraceae (II)*	≈	≈	≈	↑
*Lactobacillus (I)*	↑	↑	≈	↑
*Lactobacillus (II)*	—	—	≈	—
*Lactococcus*	↓	↓	↓	↓
Mogibacteriaceae (I) *	↑	≈	≈	≈
Mogibacteriaceae (II) *	↑	↑	↑	↑
Mucispirillum schaedleri *	↓	↓	↓	↓
*Muribaculaceae*	↑	↑	↑	↑
*Oscillospira*	↓	↓	↓	↓
*Parabacteroides gordonii*	≈	≈	≈	≈
*Peptococcaceae*	↓	↓	↓	↓
*Peptostreptococcaceae*	—	—	—	≈
rc4 4 *	↑	↑	↑	↑
*RF32*	↑	↑	↑	↑
*RF39*	≈	↑	≈	≈
*Rikenellaceae*	↑	↑	↑	↑
Roseburia *	—	—	—	↑
*Ruminococcaceae (I)*	≈	≈	≈	≈
Ruminococcaceae (II) *	≈	≈	≈	↓
*Ruminococcus gnavus*	↓	↓	↓	↓
*Ruminococcus (Lachnospiraceae)*	≈	≈	≈	≈
*Ruminococcus (Ruminococcaceae)*	≈	≈	↑	≈
*Streptococcus*	↓	↓	↓	↓
Sutterella *	↑	↑	↑	↑

* Bacteria significant amongst the pulse-based diet groups only according to LEfSe. “↑” in green indicates a statistically significant increase in abundance; “↓” in red—a decrease; “≈” in yellow—no statistical changes; and “—” indicates absence in respective pulse-type group.

**Table 4:** Summary of the pulse-induced gut microbial ecosystem.

Eco-Groups	Microbial Composition
Pulse-enhanced	*Allobaculum* *Bacteroides acidifaciens* *Butyricicoccus pullicaecorum* *Mogibacteriaceae (II)* *Muribaculaceae* *rc4 4 (Peptococcaceae)* *RF32 (Alphaproteobacteria)* *Rikenellaceae* *Sutterella*
Pulse-suppressed	*Christensenellaceae* *Clostridium methylpentosum* *Dorea* *Lactococcus* *Mucispirillum schaedleri* *Oscillospira* *Peptococcaceae* *Ruminococcus gnavus* *Streptococcus*
Pulse-indifferent	*Adlercreutzia* *Bilophila* *Clostridiales (I)* *Clostridium hathewayi* *Coprococcus* *Desulfovibrionaceae* *Enterobacteriaceae* *Erysipelotrichaceae* *F16* *Parabacteroides gordonii* *Ruminococcaceae (I)* *Ruminococcus (Lachnospiraceae).*

## Supplementary Materials

The following are available online at https://www.mdpi.com/article/10.3390/nu13113992/s1, Figure S1: PICRUSt2 results indicating differential pathways between the pulse-free and pulse-based diet groups; Table S1: Pairwise comparison of bacterial composition at phylum level across the diet groups.
