# Supplementary material for: Compositional Changes of the High-Fat Diet-Induced Gut Microbiota upon Consumption of Common Pulses
Source: Nutrients. 2021 Nov 9;13(11):3992. doi: 10.3390/nu13113992 (PMC8625176; doi:10.3390/nu13113992)
Supplement: Supplementary file 1 [file nutrients-13-03992-s001.zip › nutrients-1420067-supplementary.pdf]

Supplemental Materials

Table S1. Pairwise comparison of bacterial composition at phylum level across the diet groups

Taxon 1	Taxon 2	Correlation	*p*-value
Adlercreutzia	*Bacteroides acidifaciens*	-0.5019	0.0198
Adlercreutzia	*Mogibacteriaceae (I)*	-0.4819	0.0099
Adlercreutzia	*Muribaculaceae*	-0.4097	0.0297
Akkermansia muciniphila	*Bacteroidales*	0.4769	0.0198
Akkermansia muciniphila	*Bacteroides acidifaciens*	0.4489	0.0297
Akkermansia muciniphila	*F16*	-0.5229	0.0099
Akkermansia muciniphila	*rc4 4*	0.4385	0.0198
Allobaculum	*Anaerotruncus*	-0.5972	0.0099
Allobaculum	*Bacteroidales*	0.4989	0.0099
Allobaculum	*Bacteroides*	-0.6901	0.0099
Allobaculum	*Bacteroides acidifaciens*	0.6969	0.0099
Allobaculum	*Butyricicoccus pullicaecorum*	0.6164	0.0099
Allobaculum	*Christensenellaceae*	-0.6602	0.0099
Allobaculum	*Clostridiales (II)*	-0.6417	0.0198
Allobaculum	*Clostridium methylpentosum*	-0.665	0.0099
Allobaculum	*Coprococcus*	-0.6128	0.0099
Allobaculum	*Desulfovibrionaceae*	-0.5851	0.0099
Allobaculum	*Lactobacillus*	0.5286	0.0198
Allobaculum	*Mogibacteriaceae (II)*	0.4387	0.0198
Allobaculum	*Mucispirillum schaedleri*	-0.6859	0.0099
Allobaculum	*Muribaculaceae*	0.6225	0.0198
Allobaculum	*Oscillospira*	-0.8149	0.0099
Allobaculum	*Peptococcaceae*	-0.6891	0.0099
Allobaculum	*rc4 4*	0.6133	0.0099
Allobaculum	*RF32*	0.5875	0.0099
Allobaculum	*RF39*	0.4504	0.0099
Allobaculum	*Rikenellaceae*	0.7471	0.0099
Allobaculum	*Ruminococcaceae (I)*	-0.7728	0.0198
Allobaculum	*Ruminococcaceae (II)*	-0.6007	0.0297
Allobaculum	*Ruminococcus gnavus*	-0.7664	0.0099
Allobaculum	*Sutterella*	0.7814	0.0099
Anaerotruncus	*Allobaculum*	-0.5972	0.0099
Anaerotruncus	*Bacteroides*	0.6121	0.0099

Table S1. Pairwise comparison of bacterial composition at phylum level across the diet groups

Taxon 1	Taxon 2	Correlation	*p*-value
Anaerotruncus	*Bacteroides acidifaciens*	-0.4836	0.0396
Anaerotruncus	*Bilophila*	0.7033	0.0099
Anaerotruncus	*Christensenellaceae*	0.695	0.0099
Anaerotruncus	*Clostridiales (II)*	0.6543	0.0297
Anaerotruncus	*Clostridium methylpentosum*	0.6913	0.0099
Anaerotruncus	*Lactococcus*	0.4505	0.0198
Anaerotruncus	*Mucispirillum schaedleri*	0.6487	0.0099
Anaerotruncus	*Oscillospira*	0.7022	0.0099
Anaerotruncus	*Parabacteroides gordonii*	0.5829	0.0198
Anaerotruncus	*Peptococcaceae*	0.7029	0.0099
Anaerotruncus	*rc4 4*	-0.4664	0.0099
Anaerotruncus	*RF32*	-0.5394	0.0099
Anaerotruncus	*Rikenellaceae*	-0.5739	0.0396
Anaerotruncus	*Ruminococcaceae (I)*	0.5555	0.0396
Anaerotruncus	*Ruminococcaceae (II)*	0.6048	0.0297
Anaerotruncus	*Ruminococcus gnavus*	0.6821	0.0099
Anaerotruncus	*Streptococcus*	0.5456	0.0099
Anaerotruncus	*Sutterella*	-0.622	0.0099
Bacteroidales	*Akkermansia muciniphila*	0.4769	0.0198
Bacteroidales	*Allobaculum*	0.4989	0.0099
Bacteroidales	*Bacteroides acidifaciens*	0.6714	0.0099
Bacteroidales	*Butyricicoccus pullicaecorum*	0.5549	0.0099
Bacteroidales	*Clostridiales (II)*	-0.6171	0.0396
Bacteroidales	*Lactococcus*	-0.4184	0.0099
Bacteroidales	*Mucispirillum schaedleri*	-0.5242	0.0099
Bacteroidales	*Muribaculaceae*	0.7358	0.0099
Bacteroidales	*rc4 4*	0.472	0.0099
Bacteroidales	*RF32*	0.5945	0.0099
Bacteroidales	*Rikenellaceae*	0.5491	0.0099
Bacteroidales	*Sutterella*	0.6197	0.0099
Bacteroides	*Allobaculum*	-0.6901	0.0099
Bacteroides	*Anaerotruncus*	0.6121	0.0099
Bacteroides	*Bacteroides acidifaciens*	-0.7297	0.0099
Bacteroides	*Bilophila*	0.5469	0.0396

Table S1. Pairwise comparison of bacterial composition at phylum level across the diet groups

Taxon 1	Taxon 2	Correlation	*p*-value
Bacteroides	*Butyricicoccus pullicaecorum*	-0.5548	0.0099
Bacteroides	*Christensenellaceae*	0.6673	0.0099
Bacteroides	*Clostridium methylpentosum*	0.5067	0.0099
Bacteroides	*Lactococcus*	0.4501	0.0297
Bacteroides	*Mogibacteriaceae (II)*	-0.4732	0.0396
Bacteroides	*Mucispirillum schaedleri*	0.626	0.0099
Bacteroides	*Oscillospira*	0.7179	0.0297
Bacteroides	*Peptococcaceae*	0.7086	0.0297
Bacteroides	*rc4 4*	-0.7371	0.0198
Bacteroides	*RF32*	-0.5772	0.0198
Bacteroides	*Rikenellaceae*	-0.6628	0.0099
Bacteroides	*Ruminococcus gnavus*	0.737	0.0099
Bacteroides	*Streptococcus*	0.4986	0.0198
Bacteroides	*Sutterella*	-0.7186	0.0099
Bacteroides acidifaciens	*Adlercreutzia*	-0.5019	0.0198
Bacteroides acidifaciens	*Akkermansia muciniphila*	0.4489	0.0297
Bacteroides acidifaciens	*Allobaculum*	0.6969	0.0099
Bacteroides acidifaciens	*Anaerotruncus*	-0.4836	0.0396
Bacteroides acidifaciens	*Bacteroidales*	0.6714	0.0099
Bacteroides acidifaciens	*Bacteroides*	-0.7297	0.0099
Bacteroides acidifaciens	*Bilophila*	-0.474	0.0396
Bacteroides acidifaciens	*Butyricicoccus pullicaecorum*	0.5836	0.0099
Bacteroides acidifaciens	*Christensenellaceae*	-0.4859	0.0099
Bacteroides acidifaciens	*Clostridiales (I)*	-0.5447	0.0495
Bacteroides acidifaciens	*Clostridiales (II)*	1	0.0297
Bacteroides acidifaciens	*Clostridium methylpentosum*	-0.6347	0.0099
Bacteroides acidifaciens	*Desulfovibrionaceae*	-0.4005	0.0099
Bacteroides acidifaciens	*F16*	-0.5527	0.0099
Bacteroides acidifaciens	*Lachnospiraceae (I)*	-0.6692	0.0297
Bacteroides acidifaciens	*Lactococcus*	-0.6788	0.0099
Bacteroides acidifaciens	*Mogibacteriaceae (II)*	0.7584	0.0099
Bacteroides acidifaciens	*Mucispirillum schaedleri*	-0.6543	0.0099
Bacteroides acidifaciens	*Muribaculaceae*	0.7002	0.0297
Bacteroides acidifaciens	*Oscillospira*	-0.6911	0.0297

Table S1. Pairwise comparison of bacterial composition at phylum level across the diet groups

Taxon 1	Taxon 2	Correlation	*p*-value
Bacteroides acidifaciens	*Peptococcaceae*	-0.7333	0.0099
Bacteroides acidifaciens	*rc4 4*	0.802	0.0099
Bacteroides acidifaciens	*RF32*	0.9205	0.0099
Bacteroides acidifaciens	*Rikenellaceae*	0.8036	0.0099
Bacteroides acidifaciens	*Ruminococcus gnavus*	-0.7898	0.0198
Bacteroides acidifaciens	*Streptococcus*	-0.5349	0.0099
Bacteroides acidifaciens	*Sutterella*	0.8574	0.0099
Bilophila	*Anaerotruncus*	0.7033	0.0099
Bilophila	*Bacteroides*	0.5469	0.0396
Bilophila	*Bacteroides acidifaciens*	-0.474	0.0396
Bilophila	*rc4 4*	-0.4771	0.0099
Bilophila	*Ruminococcus (Lachnospiraceae)*	0.406	0.0396
Bilophila	*Streptococcus*	0.4858	0.0198
Bilophila	*Sutterella*	-0.5135	0.0099
Butyricicoccus pullicaecorum	*Allobaculum*	0.6164	0.0099
Butyricicoccus pullicaecorum	*Bacteroidales*	0.5549	0.0099
Butyricicoccus pullicaecorum	*Bacteroides*	-0.5548	0.0099
Butyricicoccus pullicaecorum	*Bacteroides acidifaciens*	0.5836	0.0099
Butyricicoccus pullicaecorum	*Christensenellaceae*	-0.5198	0.0099
Butyricicoccus pullicaecorum	*Clostridiales (II)*	-0.7977	0.0198
Butyricicoccus pullicaecorum	*Clostridium methylpentosum*	-0.633	0.0099
Butyricicoccus pullicaecorum	*Dehalobacterium*	-0.6866	0.0495
Butyricicoccus pullicaecorum	*Mucispirillum schaedleri*	-0.5751	0.0099
Butyricicoccus pullicaecorum	*Muribaculaceae*	0.5862	0.0198

Table S1. Pairwise comparison of bacterial composition at phylum level across the diet groups

Taxon 1	Taxon 2	Correlation	*p*-value
Butyricicoccus pullicaecorum	*Oscillospira*	-0.62	0.0198
Butyricicoccus pullicaecorum	*Parabacteroides gordonii*	-0.4809	0.0198
Butyricicoccus pullicaecorum	*Peptococcaceae*	-0.5492	0.0099
Butyricicoccus pullicaecorum	*rc4 4*	0.436	0.0198
Butyricicoccus pullicaecorum	*RF32*	0.6163	0.0099
Butyricicoccus pullicaecorum	*RF39*	0.6112	0.0099
Butyricicoccus pullicaecorum	*Rikenellaceae*	0.6001	0.0198
Butyricicoccus pullicaecorum	*Ruminococcaceae (I)*	-0.6007	0.0198
Butyricicoccus pullicaecorum	*Ruminococcaceae (II)*	-0.6147	0.0198
Butyricicoccus pullicaecorum	*Ruminococcus gnavus*	-0.6088	0.0099
Butyricicoccus pullicaecorum	*Sutterella*	0.653	0.0099
Christensenellaceae	*Allobaculum*	-0.6602	0.0099
Christensenellaceae	*Anaerotruncus*	0.695	0.0099
Christensenellaceae	*Bacteroides*	0.6673	0.0099
Christensenellaceae	*Bacteroides acidifaciens*	-0.4859	0.0099
Christensenellaceae	*Butyricicoccus pullicaecorum*	-0.5198	0.0099
Christensenellaceae	*Clostridium methylpentosum*	0.7227	0.0099
Christensenellaceae	*Coprococcus*	0.5055	0.0396
Christensenellaceae	*Dehalobacterium*	0.7359	0.0495
Christensenellaceae	*Dorea*	0.4754	0.0099
Christensenellaceae	*Lactococcus*	0.4332	0.0198
Christensenellaceae	*Mucispirillum schaedleri*	0.6366	0.0099
Christensenellaceae	*Oscillospira*	0.7229	0.0198
Christensenellaceae	*Parabacteroides gordonii*	0.4699	0.0198

Table S1. Pairwise comparison of bacterial composition at phylum level across the diet groups

Taxon 1	Taxon 2	Correlation	*p*-value
Christensenellaceae	*Peptococcaceae*	0.6144	0.0099
Christensenellaceae	*rc4 4*	-0.4383	0.0099
Christensenellaceae	*RF32*	-0.5837	0.0099
Christensenellaceae	*Rikenellaceae*	-0.6868	0.0099
Christensenellaceae	*Ruminococcaceae (II)*	0.5596	0.0297
Christensenellaceae	*Ruminococcus gnavus*	0.6767	0.0099
Christensenellaceae	*Streptococcus*	0.4039	0.0099
Christensenellaceae	*Sutterella*	-0.6203	0.0099
Clostridiales (I)	*Bacteroides acidifaciens*	-0.5447	0.0495
Clostridiales (I)	*Sutterella*	-0.4444	0.0495
Clostridiales (II)	*Allobaculum*	-0.6417	0.0198
Clostridiales (II)	*Anaerotruncus*	0.6543	0.0297
Clostridiales (II)	*Bacteroidales*	-0.6171	0.0396
Clostridiales (II)	*Bacteroides acidifaciens*	1	0.0297
Clostridiales (II)	*Butyricicoccus pullicaecorum*	-0.7977	0.0198
Clostridiales (II)	*Mucispirillum schaedleri*	0.6475	0.0396
Clostridiales (II)	*rc4 4*	-0.6854	0.0198
Clostridiales (II)	*RF32*	-0.6371	0.0297
Clostridiales (II)	*RF39*	-0.6351	0.0198
Clostridiales (II)	*Rikenellaceae*	-0.7392	0.0396
Clostridiales (II)	*Streptococcus*	0.6597	0.0198
Clostridiales (II)	*Sutterella*	-0.8571	0.0198
Clostridium colinum	*Roseburia*	0.7085	0.0099
Clostridium hathewayi	*Desulfovibrionaceae*	0.5141	0.0099
Clostridium hathewayi	*Muribaculaceae*	-0.5008	0.0198
Clostridium (I)	*Muribaculaceae*	-0.442	0.0198
Clostridium (II)	*Dorea*	-0.4974	0.0099
Clostridium (II)	*Ruminococcus (Ruminococcaceae)*	0.6413	0.0099
Clostridium methylpentosum	*Allobaculum*	-0.665	0.0099
Clostridium methylpentosum	*Anaerotruncus*	0.6913	0.0099
Clostridium methylpentosum	*Bacteroides*	0.5067	0.0099
Clostridium methylpentosum	*Bacteroides acidifaciens*	-0.6347	0.0099
Clostridium methylpentosum	*Butyricicoccus pullicaecorum*	-0.633	0.0099

Table S1. Pairwise comparison of bacterial composition at phylum level across the diet groups

Taxon 1	Taxon 2	Correlation	*p*-value
Clostridium methylpentosum	*Christensenellaceae*	0.7227	0.0099
Clostridium methylpentosum	*Desulfovibrionaceae*	0.4204	0.0099
Clostridium methylpentosum	*Lactococcus*	0.4939	0.0198
Clostridium methylpentosum	*Mucispirillum schaedleri*	0.5571	0.0099
Clostridium methylpentosum	*Muribaculaceae*	-0.5136	0.0495
Clostridium methylpentosum	*Oscillospira*	0.6992	0.0099
Clostridium methylpentosum	*Parabacteroides gordonii*	0.5318	0.0495
Clostridium methylpentosum	*Peptococcaceae*	0.573	0.0297
Clostridium methylpentosum	*rc4 4*	-0.4652	0.0099
Clostridium methylpentosum	*RF32*	-0.5764	0.0099
Clostridium methylpentosum	*RF39*	-0.4129	0.0495
Clostridium methylpentosum	*Rikenellaceae*	-0.7087	0.0099
Clostridium methylpentosum	*Ruminococcaceae (II)*	0.5615	0.0396
Clostridium methylpentosum	*Ruminococcus gnavus*	0.6431	0.0099
Clostridium methylpentosum	*Streptococcus*	0.5758	0.0099
Clostridium methylpentosum	*Sutterella*	-0.6956	0.0099
Coprococcus	*Allobaculum*	-0.6128	0.0099
Coprococcus	*Christensenellaceae*	0.5055	0.0396
Coprococcus	*Desulfovibrionaceae*	0.6027	0.0198
Coprococcus	*Dorea*	0.4412	0.0297
Coprococcus	*Mucispirillum schaedleri*	0.5846	0.0297
Coprococcus	*Rikenellaceae*	-0.4904	0.0495
Coprococcus	*Ruminococcus (Lachnospiraceae)*	0.4657	0.0396
Coprococcus	*Sutterella*	-0.5118	0.0198
Dehalobacterium	*Butyricicoccus pullicaecorum*	-0.6866	0.0495
Dehalobacterium	*Christensenellaceae*	0.7359	0.0495
Dehalobacterium	*Streptococcus*	0.6254	0.0495
Desulfovibrionaceae	*Allobaculum*	-0.5851	0.0099
Desulfovibrionaceae	*Bacteroides acidifaciens*	-0.4005	0.0099
Desulfovibrionaceae	*Clostridium hathewayi*	0.5141	0.0099
Desulfovibrionaceae	*Clostridium methylpentosum*	0.4204	0.0099
Desulfovibrionaceae	*Coprococcus*	0.6027	0.0198
Desulfovibrionaceae	*Mucispirillum schaedleri*	0.524	0.0099

Table S1. Pairwise comparison of bacterial composition at phylum level across the diet groups

Taxon 1	Taxon 2	Correlation	*p*-value
Desulfovibrionaceae	*Oscillospira*	0.5906	0.0297
Desulfovibrionaceae	*RF39*	-0.4195	0.0297
Desulfovibrionaceae	*Rikenellaceae*	-0.4792	0.0099
Desulfovibrionaceae	*Ruminococcaceae (I)*	0.8177	0.0198
Desulfovibrionaceae	*Ruminococcaceae (II)*	0.5364	0.0198
Desulfovibrionaceae	*Ruminococcus gnavus*	0.4909	0.0198
Dorea	*Christensenellaceae*	0.4754	0.0099
Dorea	*Clostridium (II)*	-0.4974	0.0099
Dorea	*Coprococcus*	0.4412	0.0297
Dorea	*Mucispirillum schaedleri*	0.5073	0.0099
Dorea	*Oscillospira*	0.4715	0.0495
Dorea	*Peptococcaceae*	0.5028	0.0297
Dorea	*RF32*	-0.4313	0.0099
Dorea	*Rikenellaceae*	-0.4171	0.0198
Dorea	*Ruminococcus gnavus*	0.4663	0.0396
Dorea	*Sutterella*	-0.4438	0.0297
Enterobacteriaceae	*Ruminococcaceae (II)*	0.4495	0.0099
Enterobacteriaceae	*Ruminococcus (Ruminococcaceae)*	0.447	0.0198
Erysipelotrichaceae	*Lachnospiraceae (II)*	-0.5727	0.0495
Erysipelotrichaceae	*Ruminococcaceae (I)*	-0.5415	0.0396
F16	*Akkermansia muciniphila*	-0.5229	0.0099
F16	*Bacteroides acidifaciens*	-0.5527	0.0099
F16	*Lactobacillus*	-0.4591	0.0099
F16	*Muribaculaceae*	-0.501	0.0099
Lachnospiraceae (I)	*Bacteroides acidifaciens*	-0.6692	0.0297
Lachnospiraceae (I)	*rc4 4*	-0.5018	0.0099
Lachnospiraceae (II)	*Erysipelotrichaceae*	-0.5727	0.0495
Lachnospiraceae (II)	*rc4 4*	-0.5261	0.0396
Lachnospiraceae (II)	*Ruminococcus (Lachnospiraceae)*	0.5788	0.0099
Lactobacillus	*Allobaculum*	0.5286	0.0198
Lactobacillus	*F16*	-0.4591	0.0099
Lactobacillus	*Oscillospira*	-0.6672	0.0198

Table S1. Pairwise comparison of bacterial composition at phylum level across the diet groups

Taxon 1	Taxon 2	Correlation	*p*-value
Lactobacillus	*Ruminococcus gnavus*	-0.5506	0.0297
Lactobacillus	*Ruminococcus (Ruminococcaceae)*	-0.6606	0.0198
Lactobacillus	*Sutterella*	0.4253	0.0396
Lactococcus	*Anaerotruncus*	0.4505	0.0198
Lactococcus	*Bacteroidales*	-0.4184	0.0099
Lactococcus	*Bacteroides*	0.4501	0.0297
Lactococcus	*Bacteroides acidifaciens*	-0.6788	0.0099
Lactococcus	*Christensenellaceae*	0.4332	0.0198
Lactococcus	*Clostridium methylpentosum*	0.4939	0.0198
Lactococcus	*Mucispirillum schaedleri*	0.5349	0.0099
Lactococcus	*Muribaculaceae*	-0.6427	0.0198
Lactococcus	*rc4 4*	-0.4528	0.0099
Lactococcus	*RF32*	-0.5218	0.0099
Lactococcus	*Rikenellaceae*	-0.5777	0.0099
Lactococcus	*Ruminococcus gnavus*	0.4753	0.0099
Lactococcus	*Streptococcus*	0.4863	0.0099
Lactococcus	*Sutterella*	-0.5508	0.0099
Mogibacteriaceae (I)	*Adlercreutzia*	-0.4819	0.0099
Mogibacteriaceae (II)	*Allobaculum*	0.4387	0.0198
Mogibacteriaceae (II)	*Bacteroides*	-0.4732	0.0396
Mogibacteriaceae (II)	*Bacteroides acidifaciens*	0.7584	0.0099
Mogibacteriaceae (II)	*Muribaculaceae*	0.6559	0.0099
Mogibacteriaceae (II)	*rc4 4*	0.5009	0.0099
Mogibacteriaceae (II)	*RF32*	0.4702	0.0099
Mogibacteriaceae (II)	*Rikenellaceae*	0.5896	0.0297
Mogibacteriaceae (II)	*Ruminococcus gnavus*	-0.4378	0.0198
Mogibacteriaceae (II)	*Sutterella*	0.5853	0.0099
Mucispirillum schaedleri	*Allobaculum*	-0.6859	0.0099
Mucispirillum schaedleri	*Anaerotruncus*	0.6487	0.0099
Mucispirillum schaedleri	*Bacteroidales*	-0.5242	0.0099
Mucispirillum schaedleri	*Bacteroides*	0.626	0.0099
Mucispirillum schaedleri	*Bacteroides acidifaciens*	-0.6543	0.0099
Mucispirillum schaedleri	*Butyricicoccus pullicaecorum*	-0.5751	0.0099

Table S1. Pairwise comparison of bacterial composition at phylum level across the diet groups

Taxon 1	Taxon 2	Correlation	*p*-value
Mucispirillum schaedleri	*Christensenellaceae*	0.6366	0.0099
Mucispirillum schaedleri	*Clostridiales (II)*	0.6475	0.0396
Mucispirillum schaedleri	*Clostridium methylpentosum*	0.5571	0.0099
Mucispirillum schaedleri	*Coprococcus*	0.5846	0.0297
Mucispirillum schaedleri	*Desulfovibrionaceae*	0.524	0.0099
Mucispirillum schaedleri	*Dorea*	0.5073	0.0099
Mucispirillum schaedleri	*Lactococcus*	0.5349	0.0099
Mucispirillum schaedleri	*Muribaculaceae*	-0.5718	0.0495
Mucispirillum schaedleri	*Oscillospira*	0.775	0.0099
Mucispirillum schaedleri	*Parabacteroides gordonii*	0.4165	0.0396
Mucispirillum schaedleri	*Peptococcaceae*	0.6764	0.0198
Mucispirillum schaedleri	*rc4 4*	-0.504	0.0297
Mucispirillum schaedleri	*RF32*	-0.5706	0.0099
Mucispirillum schaedleri	*RF39*	-0.4558	0.0297
Mucispirillum schaedleri	*Rikenellaceae*	-0.624	0.0099
Mucispirillum schaedleri	*Ruminococcaceae (I)*	0.7894	0.0495
Mucispirillum schaedleri	*Ruminococcus gnavus*	0.7156	0.0099
Mucispirillum schaedleri	*Streptococcus*	0.4798	0.0099
Mucispirillum schaedleri	*Sutterella*	-0.749	0.0099
Muribaculaceae	*Adlercreutzia*	-0.4097	0.0297
Muribaculaceae	*Allobaculum*	0.6225	0.0198
Muribaculaceae	*Bacteroidales*	0.7358	0.0099
Muribaculaceae	*Bacteroides acidifaciens*	0.7002	0.0297
Muribaculaceae	*Butyricicoccus pullicaecorum*	0.5862	0.0198
Muribaculaceae	*Clostridium hathewayi*	-0.5008	0.0198
Muribaculaceae	*Clostridium (I)*	-0.442	0.0198
Muribaculaceae	*Clostridium methylpentosum*	-0.5136	0.0495
Muribaculaceae	*F16*	-0.501	0.0099
Muribaculaceae	*Lactococcus*	-0.6427	0.0198
Muribaculaceae	*Mogibacteriaceae (II)*	0.6559	0.0099
Muribaculaceae	*Mucispirillum schaedleri*	-0.5718	0.0495
Muribaculaceae	*rc4 4*	0.6779	0.0198
Muribaculaceae	*RF32*	0.7999	0.0099
Muribaculaceae	*Rikenellaceae*	0.6916	0.0297

Table S1. Pairwise comparison of bacterial composition at phylum level across the diet groups

Taxon 1	Taxon 2	Correlation	*p*-value
Muribaculaceae	*Ruminococcus gnavus*	-0.7735	0.0099
Muribaculaceae	*Sutterella*	0.8523	0.0099
Oscillospira	*Allobaculum*	-0.8149	0.0099
Oscillospira	*Anaerotruncus*	0.7022	0.0099
Oscillospira	*Bacteroides*	0.7179	0.0297
Oscillospira	*Bacteroides acidifaciens*	-0.6911	0.0297
Oscillospira	*Butyricicoccus pullicaecorum*	-0.62	0.0198
Oscillospira	*Christensenellaceae*	0.7229	0.0198
Oscillospira	*Clostridium methylpentosum*	0.6992	0.0099
Oscillospira	*Desulfovibrionaceae*	0.5906	0.0297
Oscillospira	*Dorea*	0.4715	0.0495
Oscillospira	*Lactobacillus*	-0.6672	0.0198
Oscillospira	*Mucispirillum schaedleri*	0.775	0.0099
Oscillospira	*Peptococcaceae*	0.7573	0.0495
Oscillospira	*rc4 4*	-0.7207	0.0198
Oscillospira	*RF32*	-0.5912	0.0099
Oscillospira	*RF39*	-0.6057	0.0297
Oscillospira	*Rikenellaceae*	-0.7144	0.0198
Oscillospira	*Ruminococcus gnavus*	0.8118	0.0198
Oscillospira	*Streptococcus*	0.6218	0.0198
Oscillospira	*Sutterella*	-0.845	0.0099
Parabacteroides gordonii	*Anaerotruncus*	0.5829	0.0198
Parabacteroides gordonii	*Butyricicoccus pullicaecorum*	-0.4809	0.0198
Parabacteroides gordonii	*Christensenellaceae*	0.4699	0.0198
Parabacteroides gordonii	*Clostridium methylpentosum*	0.5318	0.0495
Parabacteroides gordonii	*Mucispirillum schaedleri*	0.4165	0.0396
Peptococcaceae	*Allobaculum*	-0.6891	0.0099
Peptococcaceae	*Anaerotruncus*	0.7029	0.0099
Peptococcaceae	*Bacteroides*	0.7086	0.0297
Peptococcaceae	*Bacteroides acidifaciens*	-0.7333	0.0099
Peptococcaceae	*Butyricicoccus pullicaecorum*	-0.5492	0.0099
Peptococcaceae	*Christensenellaceae*	0.6144	0.0099
Peptococcaceae	*Clostridium methylpentosum*	0.573	0.0297
Peptococcaceae	*Dorea*	0.5028	0.0297

Table S1. Pairwise comparison of bacterial composition at phylum level across the diet groups

Taxon 1	Taxon 2	Correlation	*p*-value
Peptococcaceae	*Mucispirillum schaedleri*	0.6764	0.0198
Peptococcaceae	*Oscillospira*	0.7573	0.0495
Peptococcaceae	*rc4 4*	-0.716	0.0099
Peptococcaceae	*RF32*	-0.6533	0.0099
Peptococcaceae	*Rikenellaceae*	-0.6968	0.0198
Peptococcaceae	*Ruminococcus gnavus*	0.7785	0.0099
Peptococcaceae	*Ruminococcus (Lachnospiraceae)*	0.4304	0.0495
Peptococcaceae	*Streptococcus*	0.6544	0.0099
Peptococcaceae	*Sutterella*	-0.6967	0.0198
rc4 4	*Akkermansia muciniphila*	0.4385	0.0198
rc4 4	*Allobaculum*	0.6133	0.0099
rc4 4	*Anaerotruncus*	-0.4664	0.0099
rc4 4	*Bacteroidales*	0.472	0.0099
rc4 4	*Bacteroides*	-0.7371	0.0198
rc4 4	*Bacteroides acidifaciens*	0.802	0.0099
rc4 4	*Bilophila*	-0.4771	0.0099
rc4 4	*Butyricicoccus pullicaecorum*	0.436	0.0198
rc4 4	*Christensenellaceae*	-0.4383	0.0099
rc4 4	*Clostridiales (II)*	-0.6854	0.0198
rc4 4	*Clostridium methylpentosum*	-0.4652	0.0099
rc4 4	*Lachnospiraceae (I)*	-0.5018	0.0099
rc4 4	*Lachnospiraceae (II)*	-0.5261	0.0396
rc4 4	*Lactococcus*	-0.4528	0.0099
rc4 4	*Mogibacteriaceae (II)*	0.5009	0.0099
rc4 4	*Mucispirillum schaedleri*	-0.504	0.0297
rc4 4	*Muribaculaceae*	0.6779	0.0198
rc4 4	*Oscillospira*	-0.7207	0.0198
rc4 4	*Peptococcaceae*	-0.716	0.0099
rc4 4	*RF32*	0.7551	0.0099
rc4 4	*Rikenellaceae*	0.6339	0.0099
rc4 4	*Ruminococcaceae (I)*	-0.5975	0.0396
rc4 4	*Ruminococcus gnavus*	-0.6217	0.0099
rc4 4	*Streptococcus*	-0.489	0.0099

Table S1. Pairwise comparison of bacterial composition at phylum level across the diet groups

Taxon 1	Taxon 2	Correlation	*p*-value
rc4 4	*Sutterella*	0.7031	0.0099
RF32	*Allobaculum*	0.5875	0.0099
RF32	*Anaerotruncus*	-0.5394	0.0099
RF32	*Bacteroidales*	0.5945	0.0099
RF32	*Bacteroides*	-0.5772	0.0198
RF32	*Bacteroides acidifaciens*	0.9205	0.0099
RF32	*Butyricicoccus pullicaecorum*	0.6163	0.0099
RF32	*Christensenellaceae*	-0.5837	0.0099
RF32	*Clostridiales (II)*	-0.6371	0.0297
RF32	*Clostridium methylpentosum*	-0.5764	0.0099
RF32	*Dorea*	-0.4313	0.0099
RF32	*Lactococcus*	-0.5218	0.0099
RF32	*Mogibacteriaceae (II)*	0.4702	0.0099
RF32	*Mucispirillum schaedleri*	-0.5706	0.0099
RF32	*Muribaculaceae*	0.7999	0.0099
RF32	*Oscillospira*	-0.5912	0.0099
RF32	*Peptococcaceae*	-0.6533	0.0099
RF32	*rc4 4*	0.7551	0.0099
RF32	*Rikenellaceae*	0.862	0.0099
RF32	*Ruminococcus gnavus*	-0.588	0.0099
RF32	*Streptococcus*	-0.6317	0.0099
RF32	*Sutterella*	0.7779	0.0099
RF39	*Allobaculum*	0.4504	0.0099
RF39	*Butyricicoccus pullicaecorum*	0.6112	0.0099
RF39	*Clostridiales (II)*	-0.6351	0.0198
RF39	*Clostridium methylpentosum*	-0.4129	0.0495
RF39	*Desulfovibrionaceae*	-0.4195	0.0297
RF39	*Mucispirillum schaedleri*	-0.4558	0.0297
RF39	*Oscillospira*	-0.6057	0.0297
RF39	*Ruminococcaceae (I)*	1	0.0297
RF39	*Ruminococcus gnavus*	-0.4498	0.0099
Rikenellaceae	*Allobaculum*	0.7471	0.0099
Rikenellaceae	*Anaerotruncus*	-0.5739	0.0396
Rikenellaceae	*Bacteroidales*	0.5491	0.0099

Table S1. Pairwise comparison of bacterial composition at phylum level across the diet groups

Taxon 1	Taxon 2	Correlation	*p*-value
Rikenellaceae	*Bacteroides*	-0.6628	0.0099
Rikenellaceae	*Bacteroides acidifaciens*	0.8036	0.0099
Rikenellaceae	*Butyricicoccus pullicaecorum*	0.6001	0.0198
Rikenellaceae	*Christensenellaceae*	-0.6868	0.0099
Rikenellaceae	*Clostridiales (II)*	-0.7392	0.0396
Rikenellaceae	*Clostridium methylpentosum*	-0.7087	0.0099
Rikenellaceae	*Coprococcus*	-0.4904	0.0495
Rikenellaceae	*Desulfovibrionaceae*	-0.4792	0.0099
Rikenellaceae	*Dorea*	-0.4171	0.0198
Rikenellaceae	*Lactococcus*	-0.5777	0.0099
Rikenellaceae	*Mogibacteriaceae (II)*	0.5896	0.0297
Rikenellaceae	*Mucispirillum schaedleri*	-0.624	0.0099
Rikenellaceae	*Muribaculaceae*	0.6916	0.0297
Rikenellaceae	*Oscillospira*	-0.7144	0.0198
Rikenellaceae	*Peptococcaceae*	-0.6968	0.0198
Rikenellaceae	*rc4 4*	0.6339	0.0099
Rikenellaceae	*RF32*	0.862	0.0099
Rikenellaceae	*Ruminococcaceae (I)*	-0.729	0.0396
Rikenellaceae	*Ruminococcaceae (II)*	-0.7436	0.0396
Rikenellaceae	*Ruminococcus gnavus*	-0.784	0.0099
Rikenellaceae	*Streptococcus*	-0.4309	0.0495
Rikenellaceae	*Sutterella*	0.7562	0.0099
Roseburia	*Clostridium colinum*	0.7085	0.0099
Ruminococcaceae (I)	*Allobaculum*	-0.7728	0.0198
Ruminococcaceae (I)	*Anaerotruncus*	0.5555	0.0396
Ruminococcaceae (I)	*Butyricicoccus pullicaecorum*	-0.6007	0.0198
Ruminococcaceae (I)	*Desulfovibrionaceae*	0.8177	0.0198
Ruminococcaceae (I)	*Erysipelotrichaceae*	-0.5415	0.0396
Ruminococcaceae (I)	*Mucispirillum schaedleri*	0.7894	0.0495
Ruminococcaceae (I)	*rc4 4*	-0.5975	0.0396
Ruminococcaceae (I)	*RF39*	1	0.0297
Ruminococcaceae (I)	*Rikenellaceae*	-0.729	0.0396
Ruminococcaceae (I)	*Ruminococcus (Lachnospiraceae)*	0.4986	0.0396

Table S1. Pairwise comparison of bacterial composition at phylum level across the diet groups

Taxon 1	Taxon 2	Correlation	*p*-value
Ruminococcaceae (I)	*Streptococcus*	0.6585	0.0297
Ruminococcaceae (I)	*Sutterella*	-0.6546	0.0396
Ruminococcaceae (II)	*Allobaculum*	-0.6007	0.0297
Ruminococcaceae (II)	*Anaerotruncus*	0.6048	0.0297
Ruminococcaceae (II)	*Butyricicoccus pullicaecorum*	-0.6147	0.0198
Ruminococcaceae (II)	*Christensenellaceae*	0.5596	0.0297
Ruminococcaceae (II)	*Clostridium methylpentosum*	0.5615	0.0396
Ruminococcaceae (II)	*Desulfovibrionaceae*	0.5364	0.0198
Ruminococcaceae (II)	*Enterobacteriaceae*	0.4495	0.0099
Ruminococcaceae (II)	*Rikenellaceae*	-0.7436	0.0396
Ruminococcaceae (II)	*Sutterella*	-0.5098	0.0495
Ruminococcus gnavus	*Allobaculum*	-0.7664	0.0099
Ruminococcus gnavus	*Anaerotruncus*	0.6821	0.0099
Ruminococcus gnavus	*Bacteroides*	0.737	0.0099
Ruminococcus gnavus	*Bacteroides acidifaciens*	-0.7898	0.0198
Ruminococcus gnavus	*Butyricicoccus pullicaecorum*	-0.6088	0.0099
Ruminococcus gnavus	*Christensenellaceae*	0.6767	0.0099
Ruminococcus gnavus	*Clostridium methylpentosum*	0.6431	0.0099
Ruminococcus gnavus	*Desulfovibrionaceae*	0.4909	0.0198
Ruminococcus gnavus	*Dorea*	0.4663	0.0396
Ruminococcus gnavus	*Lactobacillus*	-0.5506	0.0297
Ruminococcus gnavus	*Lactococcus*	0.4753	0.0099
Ruminococcus gnavus	*Mogibacteriaceae (II)*	-0.4378	0.0198
Ruminococcus gnavus	*Mucispirillum schaedleri*	0.7156	0.0099
Ruminococcus gnavus	*Muribaculaceae*	-0.7735	0.0099
Ruminococcus gnavus	*Oscillospira*	0.8118	0.0198
Ruminococcus gnavus	*Peptococcaceae*	0.7785	0.0099
Ruminococcus gnavus	*rc4 4*	-0.6217	0.0099
Ruminococcus gnavus	*RF32*	-0.588	0.0099
Ruminococcus gnavus	*RF39*	-0.4498	0.0099
Ruminococcus gnavus	*Rikenellaceae*	-0.784	0.0099
Ruminococcus gnavus	*Streptococcus*	0.5417	0.0099
Ruminococcus gnavus	*Sutterella*	-0.7761	0.0099

Table S1. Pairwise comparison of bacterial composition at phylum level across the diet groups

Taxon 1	Taxon 2	Correlation	*p*-value
Ruminococcus (Lachnospiraceae)	*Bilophila*	0.406	0.0396
Ruminococcus (Lachnospiraceae)	*Coprococcus*	0.4657	0.0396
Ruminococcus (Lachnospiraceae)	*Lachnospiraceae (II)*	0.5788	0.0099
Ruminococcus (Lachnospiraceae)	*Peptococcaceae*	0.4304	0.0495
Ruminococcus (Lachnospiraceae)	*Ruminococcaceae (I)*	0.4986	0.0396
Ruminococcus (Ruminococcaceae)	*Clostridium (II)*	0.6413	0.0099
Ruminococcus (Ruminococcaceae)	*Enterobacteriaceae*	0.447	0.0198
Ruminococcus (Ruminococcaceae)	*Lactobacillus*	-0.6606	0.0198
Streptococcus	*Anaerotruncus*	0.5456	0.0099
Streptococcus	*Bacteroides*	0.4986	0.0198
Streptococcus	*Bacteroides acidifaciens*	-0.5349	0.0099
Streptococcus	*Bilophila*	0.4858	0.0198
Streptococcus	*Christensenellaceae*	0.4039	0.0099
Streptococcus	*Clostridiales (II)*	0.6597	0.0198
Streptococcus	*Clostridium methylpentosum*	0.5758	0.0099
Streptococcus	*Dehalobacterium*	0.6254	0.0495
Streptococcus	*Lactococcus*	0.4863	0.0099
Streptococcus	*Mucispirillum schaedleri*	0.4798	0.0099
Streptococcus	*Oscillospira*	0.6218	0.0198
Streptococcus	*Peptococcaceae*	0.6544	0.0099
Streptococcus	*rc4 4*	-0.489	0.0099
Streptococcus	*RF32*	-0.6317	0.0099
Streptococcus	*Rikenellaceae*	-0.4309	0.0495
Streptococcus	*Ruminococcaceae (I)*	0.6585	0.0297
Streptococcus	*Ruminococcus gnavus*	0.5417	0.0099
Streptococcus	*Sutterella*	-0.5752	0.0099
Sutterella	*Allobaculum*	0.7814	0.0099

Table S1. Pairwise comparison of bacterial composition at phylum level across the diet groups

Taxon 1	Taxon 2	Correlation	*p*-value
Sutterella	*Anaerotruncus*	-0.622	0.0099
Sutterella	*Bacteroidales*	0.6197	0.0099
Sutterella	*Bacteroides*	-0.7186	0.0099
Sutterella	*Bacteroides acidifaciens*	0.8574	0.0099
Sutterella	*Bilophila*	-0.5135	0.0099
Sutterella	*Butyricicoccus pullicaecorum*	0.653	0.0099
Sutterella	*Christensenellaceae*	-0.6203	0.0099
Sutterella	*Clostridiales (I)*	-0.4444	0.0495
Sutterella	*Clostridiales (II)*	-0.8571	0.0198
Sutterella	*Clostridium methylpentosum*	-0.6956	0.0099
Sutterella	*Coprococcus*	-0.5118	0.0198
Sutterella	*Dorea*	-0.4438	0.0297
Sutterella	*Lactobacillus*	0.4253	0.0396
Sutterella	*Lactococcus*	-0.5508	0.0099
Sutterella	*Mogibacteriaceae (II)*	0.5853	0.0099
Sutterella	*Mucispirillum schaedleri*	-0.749	0.0099
Sutterella	*Muribaculaceae*	0.8523	0.0099
Sutterella	*Oscillospira*	-0.845	0.0099
Sutterella	*Peptococcaceae*	-0.6967	0.0198
Sutterella	*rc4 4*	0.7031	0.0099
Sutterella	*RF32*	0.7779	0.0099
Sutterella	*Rikenellaceae*	0.7562	0.0099
Sutterella	*Ruminococcaceae (I)*	-0.6546	0.0396
Sutterella	*Ruminococcaceae (II)*	-0.5098	0.0495
Sutterella	*Ruminococcus gnavus*	-0.7761	0.0099
Sutterella	*Streptococcus*	-0.5752	0.0099

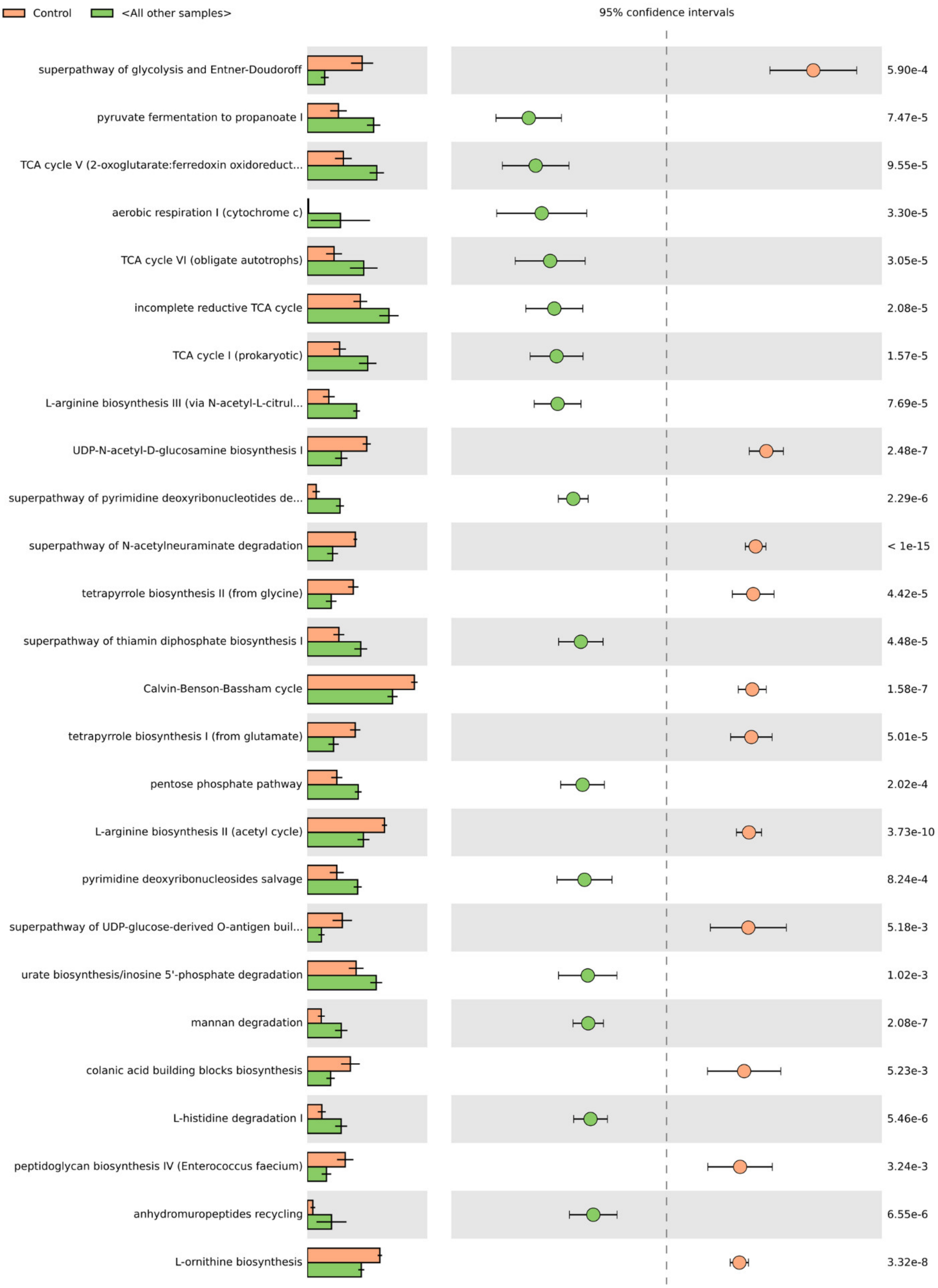
Control
<All other samples>
95% confidence intervals
superpathway of glycolysis and Entner-Doudoroff
5.90e-4
pyruvate fermentation to propanoate I
7.47e-5
TCA cycle V (2-oxoglutarate:ferredoxin oxidoreduct...
9.55e-5
aerobic respiration I (cytochrome c)
3.30e-5
TCA cycle VI (obligate autotrophs)
3.05e-5
incomplete reductive TCA cycle
2.08e-5
TCA cycle I (prokaryotic)
1.57e-5
L-arginine biosynthesis III (via N-acetyl-L-citrul...
7.69e-5
UDP-N-acetyl-D-glucosamine biosynthesis I
2.48e-7
superpathway of pyrimidine deoxyribonucleotides de...
2.29e-6
superpathway of N-acetylneuraminate degradation
< 1e-15
tetrapyrrole biosynthesis II (from glycine)
4.42e-5
superpathway of thiamin diphosphate biosynthesis I
4.48e-5
Calvin-Benson-Bassham cycle
1.58e-7
tetrapyrrole biosynthesis I (from glutamate)
5.01e-5
pentose phosphate pathway
2.02e-4
L-arginine biosynthesis II (acetyl cycle)
3.73e-10
pyrimidine deoxyribonucleosides salvage
8.24e-4
superpathway of UDP-glucose-derived O-antigen buil...
5.18e-3
urate biosynthesis/inosine 5'-phosphate degradation
1.02e-3
mannan degradation
2.08e-7
colanic acid building blocks biosynthesis
5.23e-3
L-histidine degradation I
5.46e-6
peptidoglycan biosynthesis IV (Enterococcus faecium)
3.24e-3
anhydromuropeptides recycling
6.55e-6
L-ornithine biosynthesis
3.32e-8

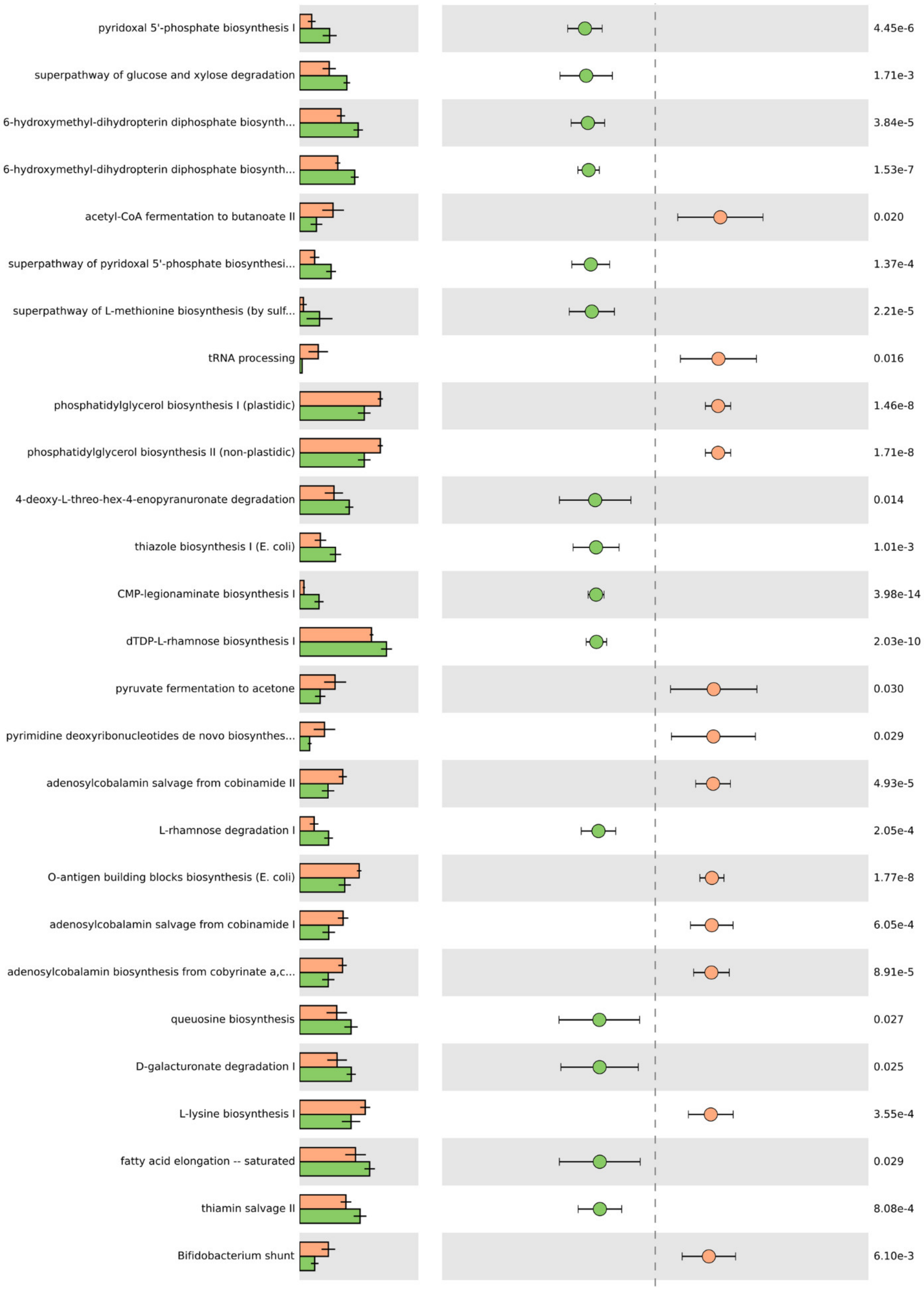

pyridoxal 5'-phosphate biosynthesis I
superpathway of glucose and xylose degradation
6-hydroxymethyl-dihydropterin diphosphate biosynth...
6-hydroxymethyl-dihydropterin diphosphate biosynth...
acetyl-CoA fermentation to butanoate II
superpathway of pyridoxal 5'-phosphate biosynthesi...
superpathway of L-methionine biosynthesis (by sulf...
tRNA processing
phosphatidylglycerol biosynthesis I (plastidic)
phosphatidylglycerol biosynthesis II (non-plastidic)
4-deoxy-L-threo-hex-4-enopyranuronate degradation
thiazole biosynthesis I (E. coli)
CMP-legionaminate biosynthesis I
dTDP-L-rhamnose biosynthesis I
pyruvate fermentation to acetone
pyrimidine deoxyribonucleotides de novo biosynthes...
adenosylcobalamin salvage from cobinamide II
L-rhamnose degradation I
O-antigen building blocks biosynthesis (E. coli)
adenosylcobalamin salvage from cobinamide I
adenosylcobalamin biosynthesis from cobyrinate a,c...
queuosine biosynthesis
D-galacturonate degradation I
L-lysine biosynthesis I
fatty acid elongation -- saturated
thiamin salvage II
Bifidobacterium shunt
4.45e-6
1.71e-3
3.84e-5
1.53e-7
0.020
1.37e-4
2.21e-5
0.016
1.46e-8
1.71e-8
0.014
1.01e-3
3.98e-14
2.03e-10
0.030
0.029
4.93e-5
2.05e-4
1.77e-8
6.05e-4
8.91e-5
0.027
0.025
3.55e-4
0.029
8.08e-4
6.10e-3

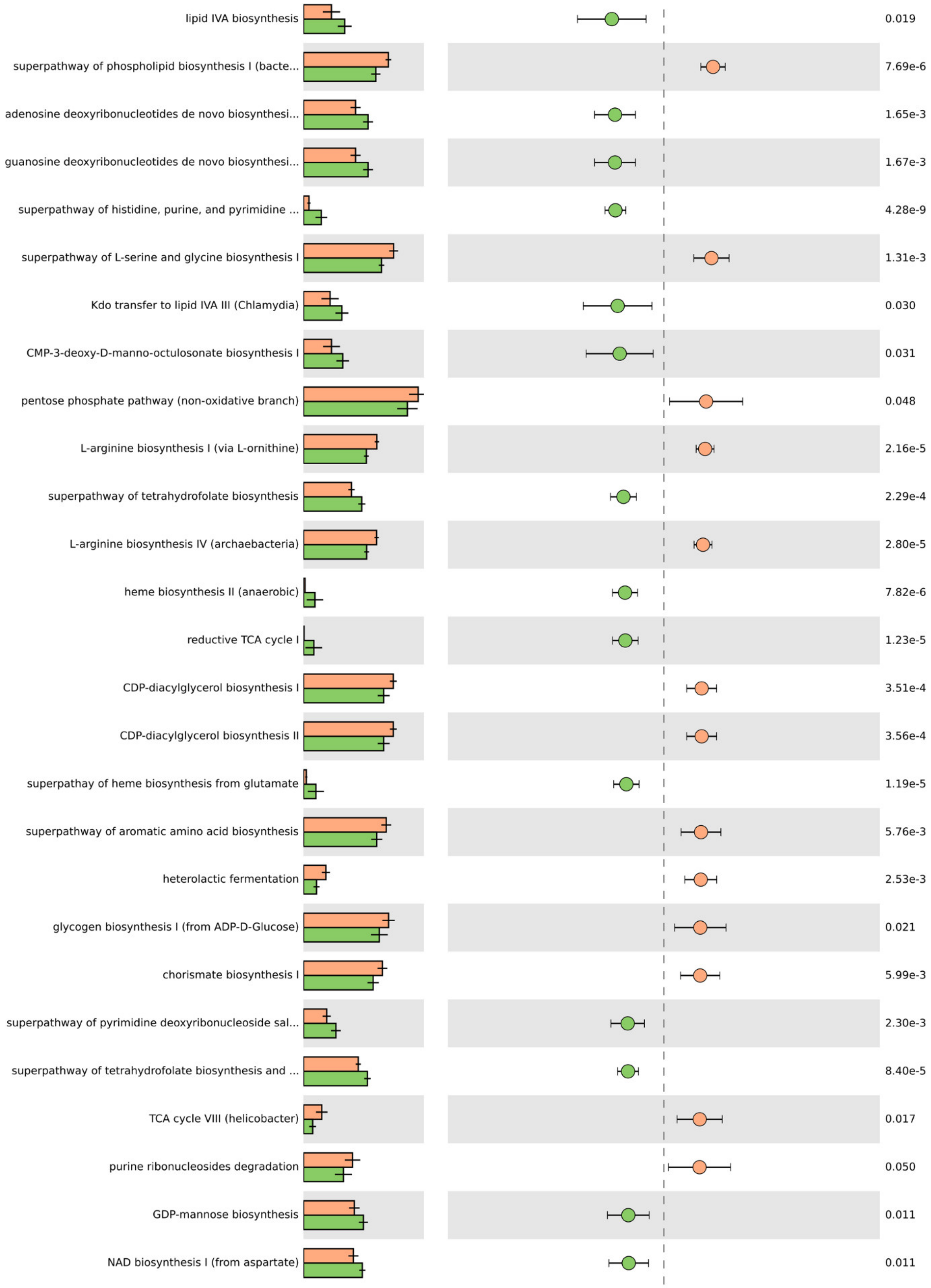
lipid IVA biosynthesis
superpathway of phospholipid biosynthesis I (bacte...
adenosine deoxyribonucleotides de novo biosynthesi...
guanosine deoxyribonucleotides de novo biosynthesi...
superpathway of histidine, purine, and pyrimidine ...
superpathway of L-serine and glycine biosynthesis I
Kdo transfer to lipid IVA III (Chlamydia)
CMP-3-deoxy-D-manno-octulosonate biosynthesis I
pentose phosphate pathway (non-oxidative branch)
L-arginine biosynthesis I (via L-ornithine)
superpathway of tetrahydrofolate biosynthesis
L-arginine biosynthesis IV (archaebacteria)
heme biosynthesis II (anaerobic)
reductive TCA cycle I
CDP-diacylglycerol biosynthesis I
CDP-diacylglycerol biosynthesis II
superpathay of heme biosynthesis from glutamate
superpathway of aromatic amino acid biosynthesis
heterolactic fermentation
glycogen biosynthesis I (from ADP-D-Glucose)
chorismate biosynthesis I
superpathway of pyrimidine deoxyribonucleoside sal...
superpathway of tetrahydrofolate biosynthesis and ...
TCA cycle VIII (helicobacter)
purine ribonucleosides degradation
GDP-mannose biosynthesis
NAD biosynthesis I (from aspartate)
0.019
7.69e-6
1.65e-3
1.67e-3
4.28e-9
1.31e-3
0.030
0.031
0.048
2.16e-5
2.29e-4
2.80e-5
7.82e-6
1.23e-5
3.51e-4
3.56e-4
1.19e-5
5.76e-3
2.53e-3
0.021
5.99e-3
2.30e-3
8.40e-5
0.017
0.050
0.011
0.011

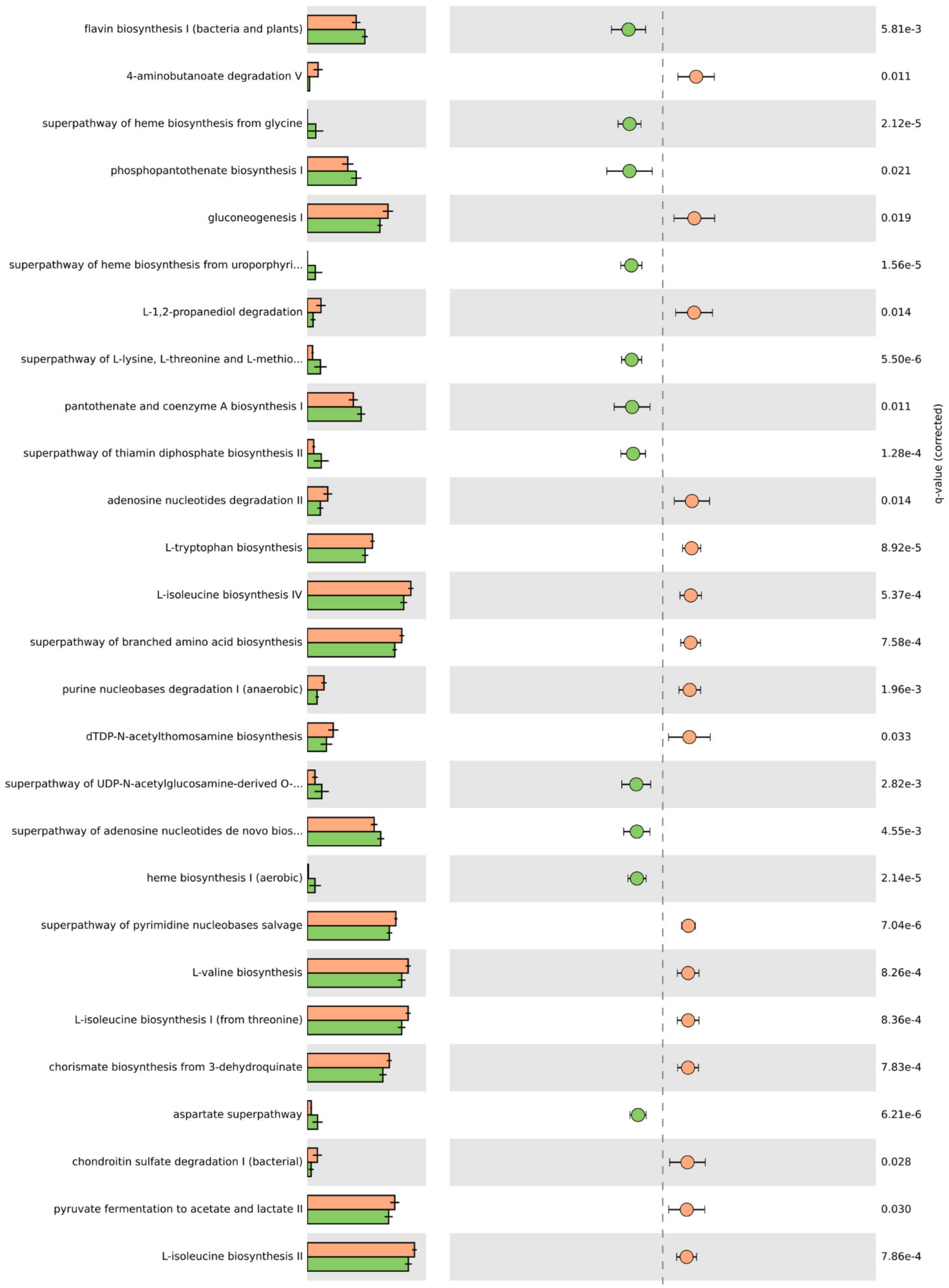

flavin biosynthesis I (bacteria and plants)
4-aminobutanoate degradation V
superpathway of heme biosynthesis from glycine
phosphopantothenate biosynthesis I
gluconeogenesis I
superpathway of heme biosynthesis from uroporphyri...
L-1,2-propanediol degradation
superpathway of L-lysine, L-threonine and L-methio...
pantothenate and coenzyme A biosynthesis I
superpathway of thiamin diphosphate biosynthesis II
adenosine nucleotides degradation II
L-tryptophan biosynthesis
L-isoleucine biosynthesis IV
superpathway of branched amino acid biosynthesis
purine nucleobases degradation I (anaerobic)
dTDP-N-acetylthomosamine biosynthesis
superpathway of UDP-N-acetylglucosamine-derived O-...
superpathway of adenosine nucleotides de novo bios...
heme biosynthesis I (aerobic)
superpathway of pyrimidine nucleobases salvage
L-valine biosynthesis
L-isoleucine biosynthesis I (from threonine)
chorismate biosynthesis from 3-dehydroquinate
aspartate superpathway
chondroitin sulfate degradation I (bacterial)
pyruvate fermentation to acetate and lactate II
L-isoleucine biosynthesis II
5.81e-3
0.011
2.12e-5
0.021
0.019
1.56e-5
0.014
5.50e-6
0.011
1.28e-4
0.014
8.92e-5
5.37e-4
7.58e-4
1.96e-3
0.033
2.82e-3
4.55e-3
2.14e-5
7.04e-6
8.26e-4
8.36e-4
7.83e-4
6.21e-6
0.028
0.030
7.86e-4
q-value (corrected)

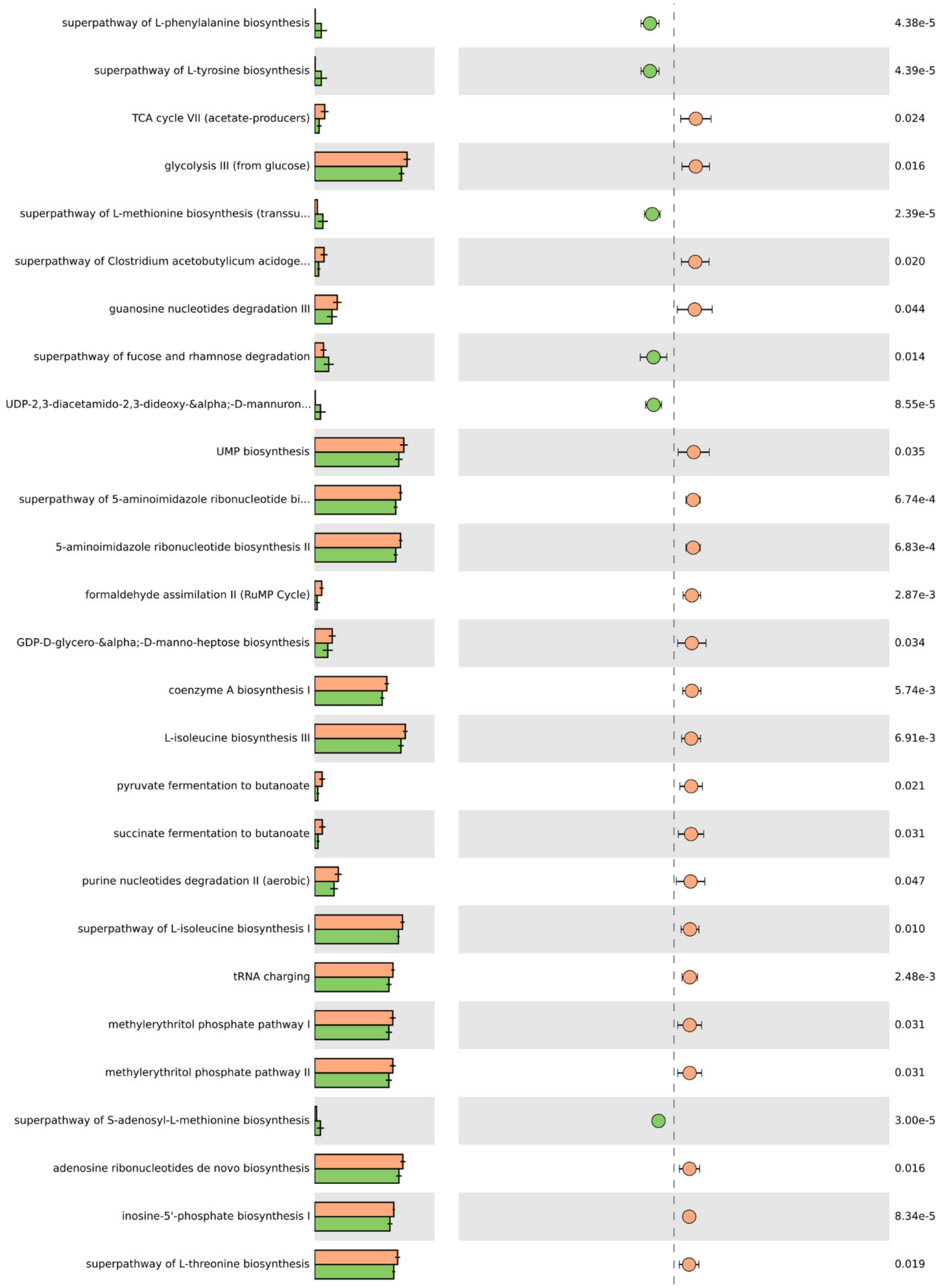
superpathway of L-phenylalanine biosynthesis
4.38e-5
superpathway of L-tyrosine biosynthesis
4.39e-5
TCA cycle VII (acetate-producers)
0.024
glycolysis III (from glucose)
0.016
superpathway of L-methionine biosynthesis (transsu...
2.39e-5
superpathway of Clostridium acetobutylicum acidoge...
0.020
guanosine nucleotides degradation III
0.044
superpathway of fucose and rhamnose degradation
0.014
UDP-2,3-diacetamido-2,3-dideoxy-α-D-mannuron...
8.55e-5
UMP biosynthesis
0.035
superpathway of 5-aminoimidazole ribonucleotide bi...
6.74e-4
5-aminoimidazole ribonucleotide biosynthesis II
6.83e-4
formaldehyde assimilation II (RuMP Cycle)
2.87e-3
GDP-D-glycero-α-D-manno-heptose biosynthesis
0.034
coenzyme A biosynthesis I
5.74e-3
L-isoleucine biosynthesis III
6.91e-3
pyruvate fermentation to butanoate
0.021
succinate fermentation to butanoate
0.031
purine nucleotides degradation II (aerobic)
0.047
superpathway of L-isoleucine biosynthesis I
0.010
tRNA charging
2.48e-3
methylerythritol phosphate pathway I
0.031
methylerythritol phosphate pathway II
0.031
superpathway of S-adenosyl-L-methionine biosynthesis
3.00e-5
adenosine ribonucleotides de novo biosynthesis
0.016
inosine-5'-phosphate biosynthesis I
8.34e-5
superpathway of L-threonine biosynthesis
0.019

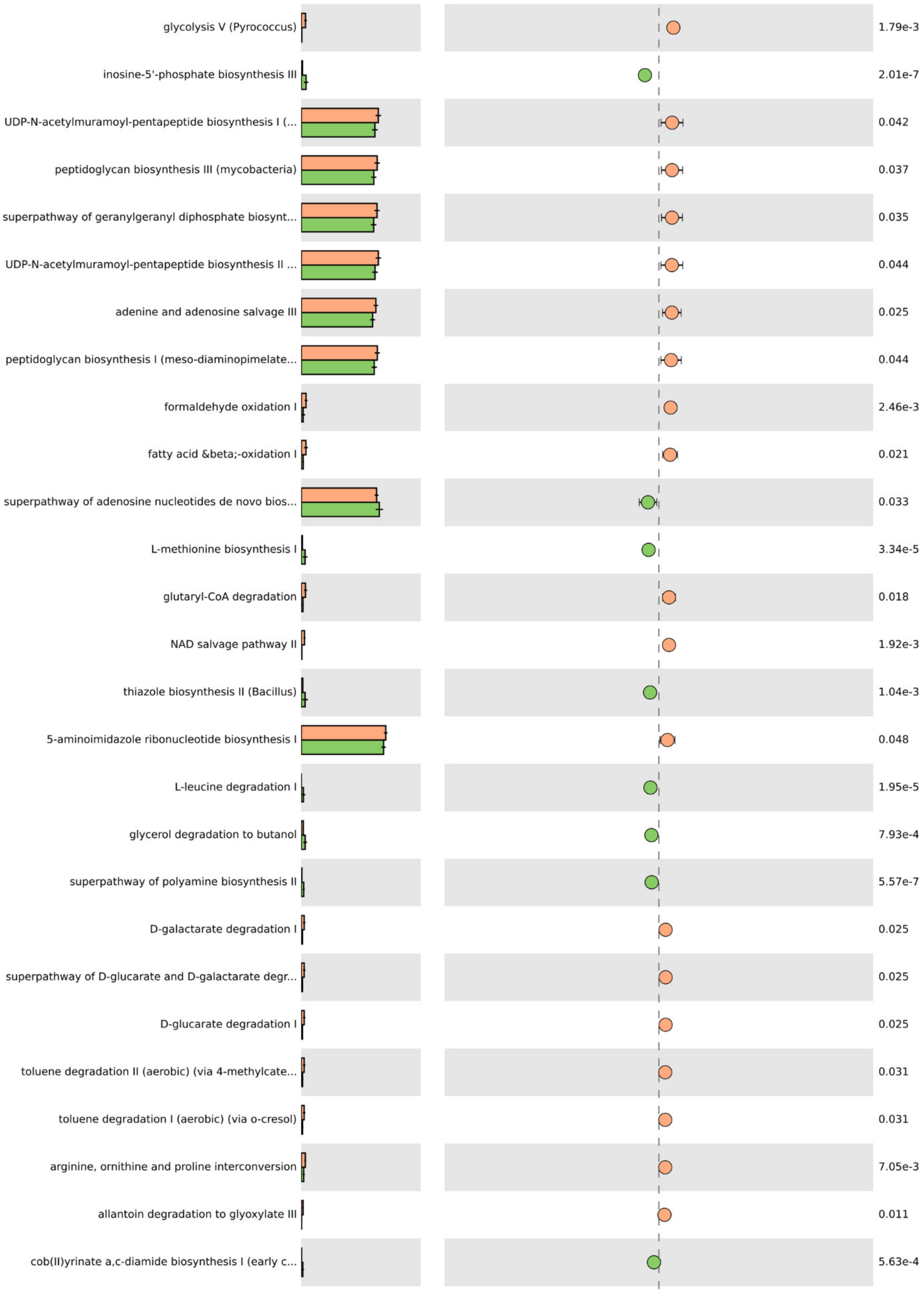
glycolysis V (Pyrococcus)
1.79e-3
inosine-5'-phosphate biosynthesis III
2.01e-7
UDP-N-acetylmuramoyl-pentapeptide biosynthesis I (...
0.042
peptidoglycan biosynthesis III (mycobacteria)
0.037
superpathway of geranylgeranyl diphosphate biosynt...
0.035
UDP-N-acetylmuramoyl-pentapeptide biosynthesis II ...
0.044
adenine and adenosine salvage III
0.025
peptidoglycan biosynthesis I (meso-diaminopimelate...
0.044
formaldehyde oxidation I
2.46e-3
fatty acid β-oxidation I
0.021
superpathway of adenosine nucleotides de novo bios...
0.033
L-methionine biosynthesis I
3.34e-5
glutaryl-CoA degradation
0.018
NAD salvage pathway II
1.92e-3
thiazole biosynthesis II (Bacillus)
1.04e-3
5-aminoimidazole ribonucleotide biosynthesis I
0.048
L-leucine degradation I
1.95e-5
glycerol degradation to butanol
7.93e-4
superpathway of polyamine biosynthesis II
5.57e-7
D-galactarate degradation I
0.025
superpathway of D-glucarate and D-galactarate degr...
0.025
D-glucarate degradation I
0.025
toluene degradation II (aerobic) (via 4-methylcate...
0.031
toluene degradation I (aerobic) (via o-cresol)
0.031
arginine, ornithine and proline interconversion
7.05e-3
allantoin degradation to glyoxylate III
0.011
cob(II)yrinate a,c-diamide biosynthesis I (early c...
5.63e-4

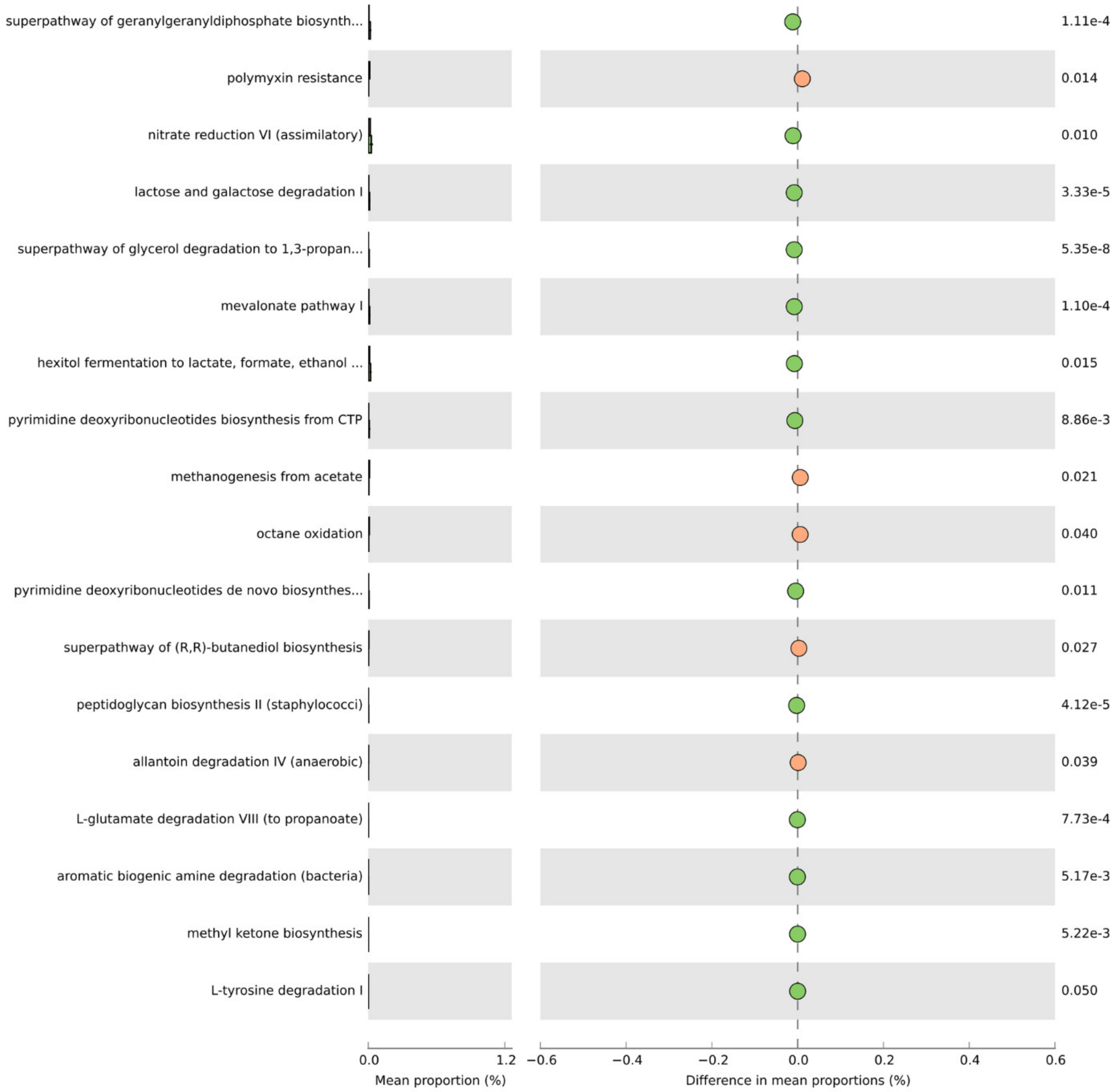

Figure S1. PICRUSt2 results indicating differential pathways between the pulse-free and pulse-supplemented diet groups. 179 significant pathways are presented here in the Extended error bar plot indicating the mean proportion of pathways assigned to each group, difference between them, and corrected *p*-value (*q*-value) of each: pulse-free diet (*Control*) is indicated in beige, while pulse-based diet samples (*All other samples*)—in green:
